# Efficacy of cognitive behavioral therapy for stimulant use disorders: a systematic review and meta-analysis

**DOI:** 10.3389/fpsyt.2025.1695702

**Published:** 2025-11-10

**Authors:** Jongtae Kim, Jaeseok Kwak, Hyunsuk Jeong, Na Jin Kim, Seung-Yup Lee, Yangsik Kim, Jangrae Kim, Sooyeon Han, Hae-ryoung Chun, Kyoung Jae Park, Soo-Bi Lee, Gyeongmin Kim, Hae Kook Lee, Hyeon Woo Yim

**Affiliations:** 1 Department of Psychiatry, Uijeongbu St. Mary’s Hospital, College of Medicine, The Catholic University of Korea, Seoul, Republic of Korea; 2 Addiction Policy Lab, College of Medicine, The Catholic University of Korea, Seoul, Republic of Korea; 3 Department of Preventive Medicine, College of Medicine, The Catholic University of Korea, Seoul, Republic of Korea; 4 Medical Library, The Catholic University of Korea, Seoul, Republic of Korea; 5 Department of Psychiatry, Eunpyeong St. Mary’s Hospital, College of Medicine, The Catholic University of Korea, Seoul, Republic of Korea; 6 Department of Psychiatry, Inha University Hospital, College of Medicine, Inha University, Incheon, Republic of Korea; 7 Department of Psychiatry, National Medical Center, Seoul, Republic of Korea; 8 Department of Psychiatry, College of Medicine, The Catholic University of Korea, Seoul, Republic of Korea; 9 Department of Social Welfare, College of Social Sciences, Chung-Ang University, Seoul, Republic of Korea; 10 Department of Social Welfare, Daejin University, Pocheon-si, Republic of Korea

**Keywords:** cognitive behavioral therapy, stimulant use disorder, methamphetamine, amphetamine, cocaine

## Abstract

**Background:**

Cognitive Behavioral Therapy (CBT) is a widely used psychosocial intervention for stimulant use disorder (SUD). However, its independent efficacy is not well established, as previous reviews often combine it with other interventions or compare it to comparators with active components. To clarify its specific contribution, this systematic review and meta-analysis aimed to determine the efficacy of standalone CBT compared to minimal-treatment controls for achieving abstinence in individuals with SUD.

**Methods:**

We conducted a systematic search of PubMed, Embase, PsycINFO, and the Cochrane Library through May 15, 2025, for randomized controlled trials (RCTs) that compared standalone CBT with minimal-treatment comparators, such as treatment-as-usual or wait-list controls, for individuals with SUD. The primary outcome was short-term stimulant abstinence. We used the Cochrane Risk of Bias 2.0 tool for risk of bias assessment and pooled odds ratios (ORs) using a random-effects model. The review protocol was registered with PROSPERO (CRD420251012327).

**Results:**

Nine RCTs met the inclusion criteria, with eight trials (849 participants) included in the meta-analysis. Standalone CBT was associated with higher odds of achieving short-term (4–24 weeks) stimulant abstinence compared to minimal-treatment controls (OR = 2.88, 95% CI = 1.08–7.70), although between-study heterogeneity was substantial (I² = 75.62%). The certainty of this evidence was rated as low using the GRADE approach, due to risk of bias and imprecision. Treatment dropout rates were similar between CBT and control groups (OR = 1.13, 95% CI = 0.67–1.91), and no CBT-related adverse events were reported.

**Conclusions:**

The findings suggest that standalone CBT may increase short-term abstinence from stimulants. However, given the low certainty of the evidence, the effect estimate should be interpreted cautiously, and more high-quality research is needed. This research was funded by the Ministry of Health and Welfare, Republic of Korea.

**Systematic Review Registration:**

PROSPERO, identifier CRD420251012327.

## 1 Introduction

Stimulant use disorder (SUD), encompassing dependence on cocaine, amphetamine, methamphetamine, and related compounds, represent a growing public health crisis worldwide (1). While historically prevalent in Western nations, SUDs are rising sharply in Asia, with record methamphetamine seizures in East and Southeast Asia highlighting the region’s escalating crisis (2, 3). This growing prevalence has been accompanied by severe medical, psychiatric, and social consequences, including psychosis, cardiovascular complications, infectious diseases (HIV and hepatitis), and elevated mortality (1). Given these widespread and severe consequences, there is an urgent need for effective and evidence-based treatment strategies.

Without approved pharmacotherapies for SUD, psychosocial interventions are the mainstay (1). In particular, among these approaches, Contingency Management (CM) has garnered the strongest evidence; it is a behavioral incentive program that rewards patients (e.g., with vouchers or prizes) for biochemical evidence of abstinence (1). However, its application is often hindered by organizational and ethical barriers (4), particularly in countries like the Republic of Korea, where stimulant use is increasing, specialized services remain underdeveloped, and stigma against addiction persists (5). Even when a psychosocial intervention has a strong evidence base, its real-world utility is limited in settings where its routine delivery is not feasible. Therefore, this situation highlights the need for more scalable and flexible treatment approaches.

In this regard, Cognitive Behavioral Therapy (CBT) represents a promising treatment option that offers a practical and scalable model of care. CBT is a structured, problem-focused psychotherapy that helps individuals identify and modify dysfunctional thoughts and behaviors through collaborative, goal-oriented techniques such as cognitive restructuring, activity scheduling, and coping-skills training (6). It can be delivered by existing healthcare providers in both individual and group settings, follows a manualized format, and does not depend on external incentives or complex program structures (7, 8). These features make CBT adaptable across a variety of clinical environments, including outpatient and community-based settings.

Although previous reviews have generally supported the efficacy of CBT, most have examined it in conjunction with other interventions, such as the Community Reinforcement Approach (CRA), or against comparison conditions that, despite being labeled as treatment-as-usual (TAU), still included active therapeutic components (1, 9, 10). As a result, the independent efficacy of CBT as a standalone treatment, particularly when compared with minimal-treatment controls, remains unclear. This represents a critical evidence gap in contexts that require immediately deployable and accessible therapies, especially where other resource-intensive alternatives are difficult to implement.

This systematic review addresses that gap by applying strict inclusion criteria for both the intervention and comparator to isolate trials comparing standalone CBT with minimal-treatment controls. This focused approach aims to clarify CBT’s independent effects, providing timely evidence for regions where practical interventions are urgently needed due to rising stimulant use and limited access to specialized care.

## 2 Methods

This systematic review followed a registered protocol (PROSPERO ID: CRD420251012327) and the Preferred Reporting Items for Systematic Reviews and Meta-Analyses (PRISMA) 2020 guidelines (11).

### 2.1 Eligibility criteria

#### 2.1.1 Population

Eligible studies included participants of any age diagnosed with SUD based on the Diagnostic and Statistical Manual of Mental Disorders (DSM) or the International Classification of Diseases (ICD) criteria or characterized by regular or dependent stimulant use. Studies focusing on recreational, non-dependent use were excluded.

#### 2.1.2 Intervention

We included studies that assessed CBT as a standalone, structured, manual-based intervention specifically targeting stimulant use. CBT could be delivered individually or in groups, in person or digitally, and added to usual care (e.g., basic counseling or medical management) if no other structured intervention was provided. Trials were eligible if CBT remained the primary focus, with any additional components (e.g., motivational enhancement) limited in scope and duration. We excluded studies that integrated other interventions [e.g., CRA (12) or motivational interviewing (13)] equally with CBT or used CBT solely as aftercare following a different primary treatment (14).

#### 2.1.3 Comparator

Eligible comparators included TAU, no-treatment conditions (e.g., wait-list or assessment-only), and attention-matched placebos that provided similar therapist contact without active therapeutic content. TAU refers to routine care without structured intervention. Studies comparing CBT to other psychosocial therapies, such as 12-step programs (15) or acceptance and commitment therapy (16), were excluded. The goal was to isolate the specific effects of CBT by including only minimal comparators. For instance, Shoptaw et al. (17) was excluded because its control group involved active components (e.g., peer counseling), exceeding the minimal-treatment criterion, despite being classified as TAU in another review (1).

#### 2.1.4 Outcomes

The primary outcome was short−term abstinence from the target stimulant, operationalized as a binary endpoint (abstinent *vs*. not abstinent). The review focused on short-term efficacy to evaluate the immediate impact of CBT as a first-line option. “Short-term” was defined as assessments conducted at each study’s designated primary endpoint; if none were specified, outcomes from the earliest post-treatment follow-up were used. Abstinence was accepted as (i) point−prevalence at the end of treatment or (ii) sustained abstinence over a prespecified continuous period (e.g., ≥ 3 consecutive weeks) during the treatment window. Biological verification (urine toxicology) was prioritized when available; a self−report was used otherwise. Analyses preferred intention-to-treat (ITT) datasets to minimize bias, classifying participants lost to follow-up as non-abstinent. When trials reported only continuous measures of stimulant use (e.g., frequency or quantity) without a compatible binary abstinence outcome, these data were not pooled in the primary meta−analysis. They were instead narratively synthesized.

Secondary outcomes included treatment dropout and adverse events. Dropout was defined as the proportion of randomized participants who did not complete the assigned intervention. If the number of completers was unavailable, dropout was estimated based on those assessed at the earliest post-treatment time point. Adverse events included any negative effects attributed to CBT.

Abstinence outcomes beyond the prespecified short-term period were excluded from the meta-analysis due to heterogeneity in follow-up durations and outcome measures, but they were narratively reviewed to explore the potential durability of treatment effects.

#### 2.1.5 Study design

Only peer-reviewed randomized controlled trials (RCTs) published in English were included. Non-randomized studies, grey literature, conference abstracts, book chapters, and protocols were excluded.

### 2.2 Information sources and search strategy

We systematically searched PubMed, Embase, PsycINFO, and Cochrane Library from inception to May 15, 2025. Reference lists of included studies and relevant reviews were also screened. Search terms for SUD and CBT were combined using “AND,” along with a sensitivity-focused RCT filter (18). No other filters or restrictions were applied. Full search strategies are detailed in **Supplementary Appendix A**.

### 2.3 Study selection

All search results were deduplicated using reference management software. Two reviewers (JK and JsK) independently screened titles/abstracts and then full texts, resolving disagreements through discussion or a third reviewer (HWY). The selection process was documented in a PRISMA 2020 flow diagram.

### 2.4 Data extraction

Two reviewers independently extracted data on study design, participants, interventions, comparators, and outcomes using a standardized, pilot-tested form, with a third reviewer resolving disagreements.

For the outcome definitions and time points, we adhered to the prespecified framework in Section 2.1.4. Accordingly, we extracted event counts and group totals for binary outcomes (abstinence and dropout) at the study’s designated primary endpoint or the earliest post-treatment follow-up, prioritizing ITT datasets. For the study where the necessary data for the meta-analysis were presented only in a figure, we extracted the numerical values using the software Plot Digitizer (19). Additionally, we reviewed all included studies for any reports of adverse events related to CBT, which were planned for narrative synthesis.

### 2.5 Risk of bias assessment

We assessed the risk of bias and publication bias for both the primary outcome (stimulant abstinence) and the secondary outcome (treatment dropout). Adverse events were narratively summarized and were not subjected to risk-of-bias assessment. Two reviewers independently assessed the risk of bias using the Cochrane Risk of Bias 2.0 (RoB2) tool (20), with a third reviewer resolving any disagreements. Reporting bias was evaluated by comparing reported outcomes with those outlined in protocols or registries. Publication bias was explored using visual inspection of a funnel plot and Egger’s test. Given the small number of studies (*k* < 10), these publication-bias analyses were considered exploratory. The results of the risk-of-bias and publication-bias assessments for the secondary outcome of treatment dropout are presented in **Supplementary Appendix C and D**.

### 2.6 Data synthesis and statistical analysis

All analyses were conducted in Stata 18.5 (StataCorp LLC, College Station, TX, USA). For dichotomous outcomes, we pooled odds ratios (ORs) with 95% confidence intervals (CIs) using a random−effects model with restricted maximum likelihood (REML), accounting for between−study heterogeneity. Analyses were primarily based on ITT data, treating participants lost to follow-up as non-abstinent. Per-protocol or completer data, when reported, were considered for sensitivity analyses. Heterogeneity was assessed with Cochran’s Q (χ²) and quantified as I²; values ≥ 50% were interpreted as substantial heterogeneity. When a single zero cell was present, we applied a continuity correction of 0.5 to all four cells to compute ORs.

### 2.7 Subgroup analyses

To investigate potential sources of the substantial heterogeneity observed in the primary analysis, we conducted several subgroup analyses.

As specified in our protocol, we performed subgroup comparisons based on two prespecified potential effect modifiers: (a) the primary stimulant type and (b) the format of CBT delivery. For the primary stimulant type, studies were categorized as focusing on either “cocaine” or “methamphetamine/amphetamine” to assess whether the efficacy of CBT varies by substance. For the delivery format, we compared studies that utilized “individual” *vs*. “group-based” CBT. A planned analysis comparing in-person *vs*. computer-assisted CBT was not performed, as only one study included in the meta-analysis used a computer-assisted format.

Given the high degree of heterogeneity, we also conducted three additional exploratory, *post hoc* subgroup analyses. First, we stratified studies by geographic region (“Asia” *vs*. “non-Asia”) to assess possible contextual influences. Second, to explore a potential dose-response effect, we conducted an analysis based on treatment intensity, categorizing studies by the number of planned CBT sessions (“≤ 8 sessions” *vs*. “> 8 sessions”). Third, to assess the influence of methodological factors on the results, we performed an analysis based on the method of outcome verification (“urine-verified” *vs*. “self-report”). We acknowledge that these *post hoc* analyses are exploratory, and their results should be interpreted with caution.

### 2.8 Sensitivity analyses

To test the robustness of the primary findings, we conducted three sensitivity analyses. First, the meta-analysis was repeated using available per-protocol/completer data to assess the impact of participant attrition and protocol deviations. Second, to evaluate the influence of outcome definition, the analysis was restricted to studies measuring point-prevalence abstinence (i.e., abstinence at the end of CBT). This outcome directly aligns with the review’s focus on immediate post-treatment effects, as opposed to sustained abstinence, which represents a broader therapeutic goal. Third, we conducted an analysis excluding studies judged to have an overall high risk of bias to evaluate whether the overall results were sensitive to the inclusion of these trials. A *post hoc* sensitivity analysis was also conducted by excluding the studies with zero events in the control group to test the stability of the results.

### 2.9 Certainty of evidence assessment

The certainty of evidence for the primary outcome (stimulant abstinence) and the secondary outcome (treatment dropout) was assessed using the GRADE approach, accounting for bias, inconsistency, indirectness, imprecision, and publication bias (21). Results were summarized in the Summary of Findings table.

## 3 Results

### 3.1 Search results

**Figure 1:**
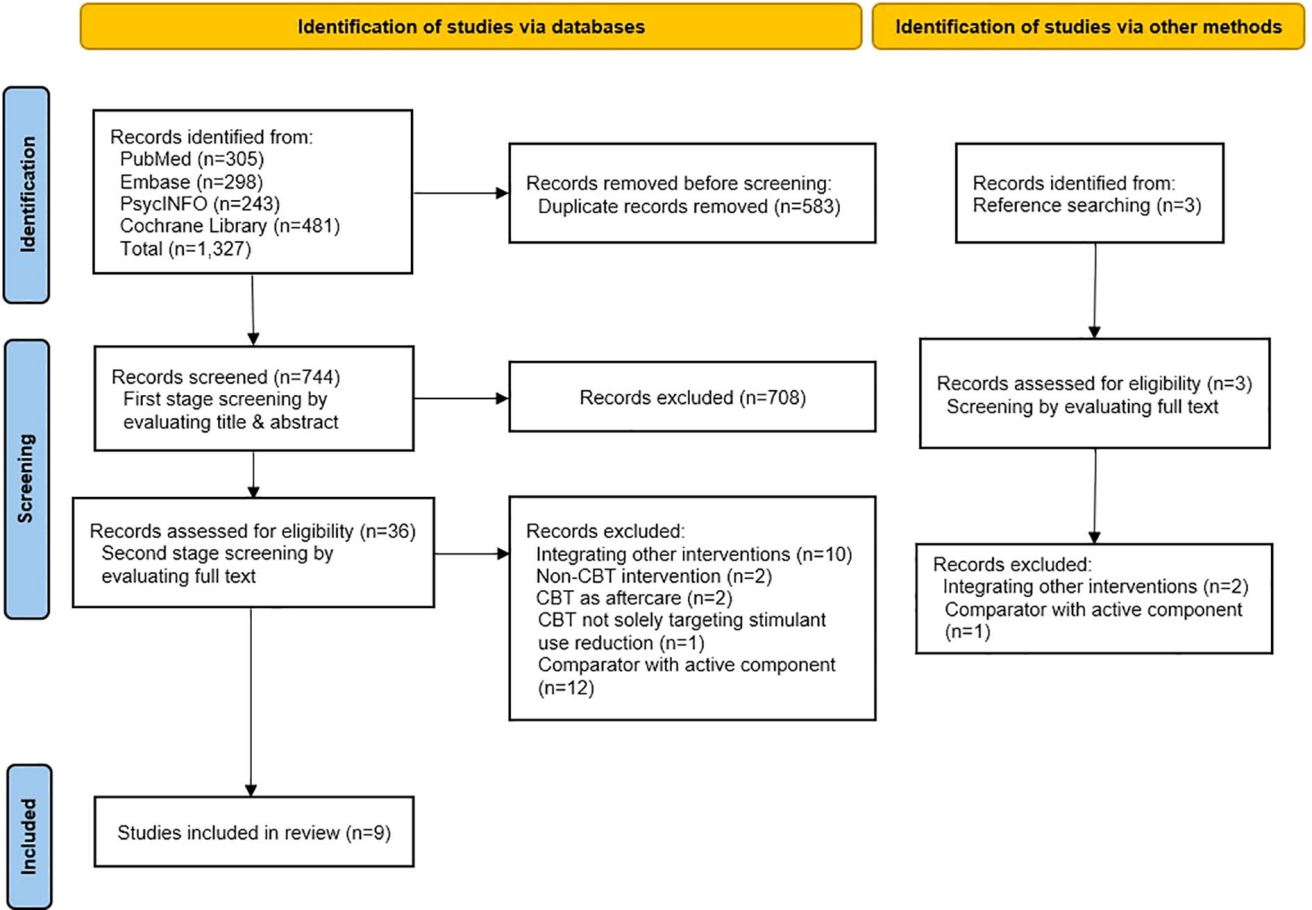
PRISMA 2020 flow diagram illustrating the study selection process.

After removing duplicates, 744 records were screened. Full-text reviews were conducted for 36 studies, along with 3 additional records from reference searching. Of these, 30 were excluded (reasons in **Figure 1**), and 9 studies met the inclusion criteria and were included in the review (22–30). Studies excluded at the full-text stage are listed in **Supplementary Appendix B**.

### 3.2 Characteristics of included studies

**Table 1** summarizes the included studies. Most (7 of 9) were conducted in Western countries. Five studies targeted cocaine, two amphetamine (23, 24), and two methamphetamine (22, 30). All were outpatient trials. Female representation ranged from 16% to 100%, with mean participant ages typically between 30 and 40. CBT was mostly delivered individually (7 studies), face-to-face (7 studies), with two studies using group (27, 28) or computer-assisted formats (25, 26). Session counts ranged from 2 to 48.

**Table 1:** Characteristics of the included studies.

Study (reference no.)	Country	Population (stimulant; mean age; female (%))	Intervention (format; no. of sessions)	Comparator	Primary outcome^a^
Alammehrjerdi et al., 2019 (22)	Iran	Methamphetamine; Not reported; 100%	Individual, face-to-face; 4 sessions	Drug education	Urine verified methamphetamine abstinence rate at the 4-week follow-up
Baker et al., 2001 (23)	Australia	Amphetamine; 31; 41%	Individual, face-to-face; 2, 4 sessions	Self-help booklet	Self-reported amphetamine abstinence rate at 6-month follow-up
Baker et al., 2005 (24)	Australia	Amphetamine; 30; 37%	Individual, face-to-face; 2, 4 sessions	Self-help booklet	Self-reported amphetamine abstinence rate at 6-month follow-up
Carroll et al., 2014 (25)	USA	Cocaine; 42; 60%	Individual, computer-assisted; 7 sessions	Standard methadone maintenance	Urine verified, number of participants attaining 3 or more weeks of continuous abstinence during 8-week treatment
Carroll et al., 2018 (26)	USA	Cocaine; 38; 26%	Individual, computer-assisted; 7 sessions	Placebo plus standard methadone maintenance	Self-reported percentage of cocaine-abstinent days per month^b^
Dursteler-MacFarland et al., 2013 (27)	Switzerland	Cocaine; 35; 41%	Group, face-to-face; 12 sessions	Placebo plus diacetylmorphine maintenance	Urine verified, number of participants attaining 3 or more weeks of continuous abstinence during 12-week treatment
Rawson et al., 2002 (28)	USA	Cocaine; 44; 49%	Group, face-to-face; 48 sessions	Standard methadone maintenance	Urine verified cocaine abstinence rate at the 17-week follow-up
Schmitz et al., 2008 (29)	USA	Cocaine; 41; 16%	Individual, face-to-face; 12 sessions	Placebo plus clinical management	Urine verified, number of participants attaining 3 or more weeks of continuous abstinence during 12-week treatment
Shakiba et al., 2018 (30)	Iran	Methamphetamine; 34; 50%	Individual, face-to-face; 16 sessions	Wait-list control	Urine verified methamphetamine abstinence rate at the 16-week follow-up

a. Primary outcome refers to the main measure of stimulant abstinence selected based on suitability for meta-analysis.

b. Since the data necessary for meta-analysis were unavailable, the study’s primary outcome is reported instead.

### 3.3 Risk of bias assessment

**Figure 2:**
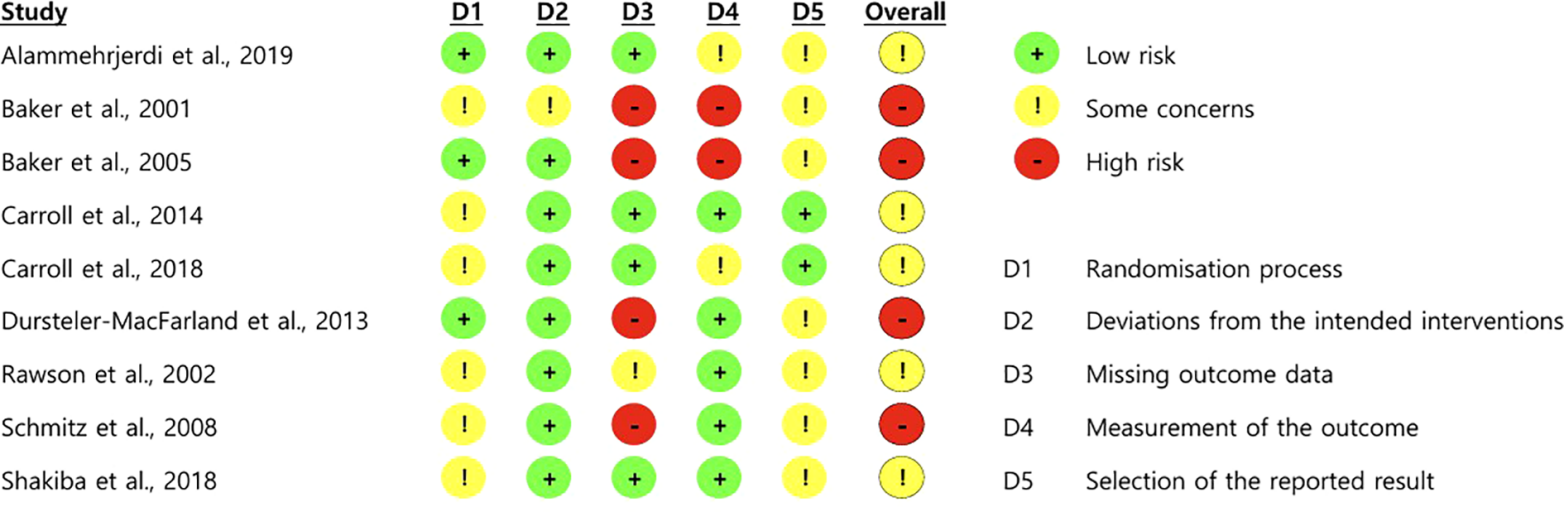
Risk of bias assessment for the primary outcome of stimulant abstinence.

Based on ITT (effect-of-assignment) analyses of the abstinence outcome used in this review, **Figure 2** summarizes RoB 2 judgments for the included RCTs. Four trials (23, 24, 27, 29) were judged to have an overall high risk of bias, primarily due to Domain 3 (missing outcome data). Reasons included non-negligible attrition without bias-robust correction, plausible outcome-dependent missingness, and treatment-arm differences in missingness. Domain 4 (measurement of the outcome) introduced additional concerns. Two trials (23, 24) were high risk because the primary endpoints depended on unblinded participant self-reports. In the case of Carroll et al. (26), the risk for a self-reported outcome was assessed as “some concerns” rather than “high” because concurrent urine toxicology screening provided partial objective verification, likely mitigating the risk of widespread inaccurate reporting. Conversely, in Alammehrjerdi et al. (22), the risk of bias in outcome measurement was judged to be some concerns, despite the use of an objective urine-verified test, because follow-up urine testing was performed only for participants who self-reported abstinence. Even with biochemical verification, conditional testing can propagate any errors in self-reports into the classification of abstinence, thereby leaving a risk of misclassification. Other domains (randomization, deviations from intended interventions, and selection of the reported result) were generally “low risk” or “some concerns.”

### 3.4 Efficacy of CBT on stimulant abstinence

**Figure 3:**
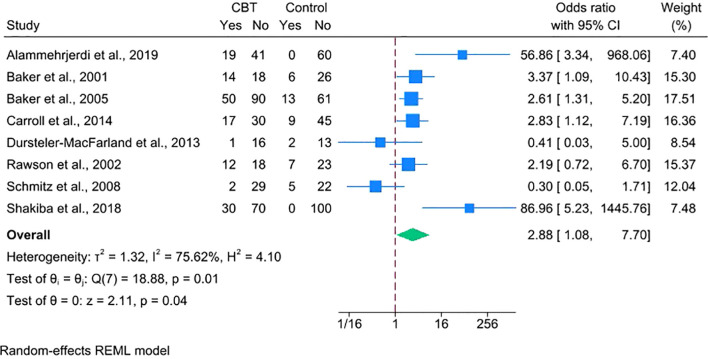
Forest plot depicting the efficacy of CBT on stimulant abstinence.

We conducted a meta-analysis of eight RCTs (N = 849; CBT = 457, control = 392), excluding Carroll et al. (26), due to the unavailability of suitable outcome data. The pooled outcome was a dichotomous measure of stimulant abstinence, defined as end-of-treatment or sustained abstinence. Using a random-effects model and ITT approach, CBT was associated with higher odds of stimulant abstinence over short-term follow-ups ranging from 4 to 24 weeks (OR = 2.88, 95% CI = 1.08–7.70, *p* = 0.04), though heterogeneity was substantial (I² = 75.62%) (**Figure 3**). While not included in the meta-analysis, Carroll et al. found that computerized CBT significantly increased days of cocaine abstinence. Overall, these findings suggest that standalone CBT increases short-term abstinence relative to minimal treatment.

### 3.5 Dropout rate and adverse events

**Figure 4:**
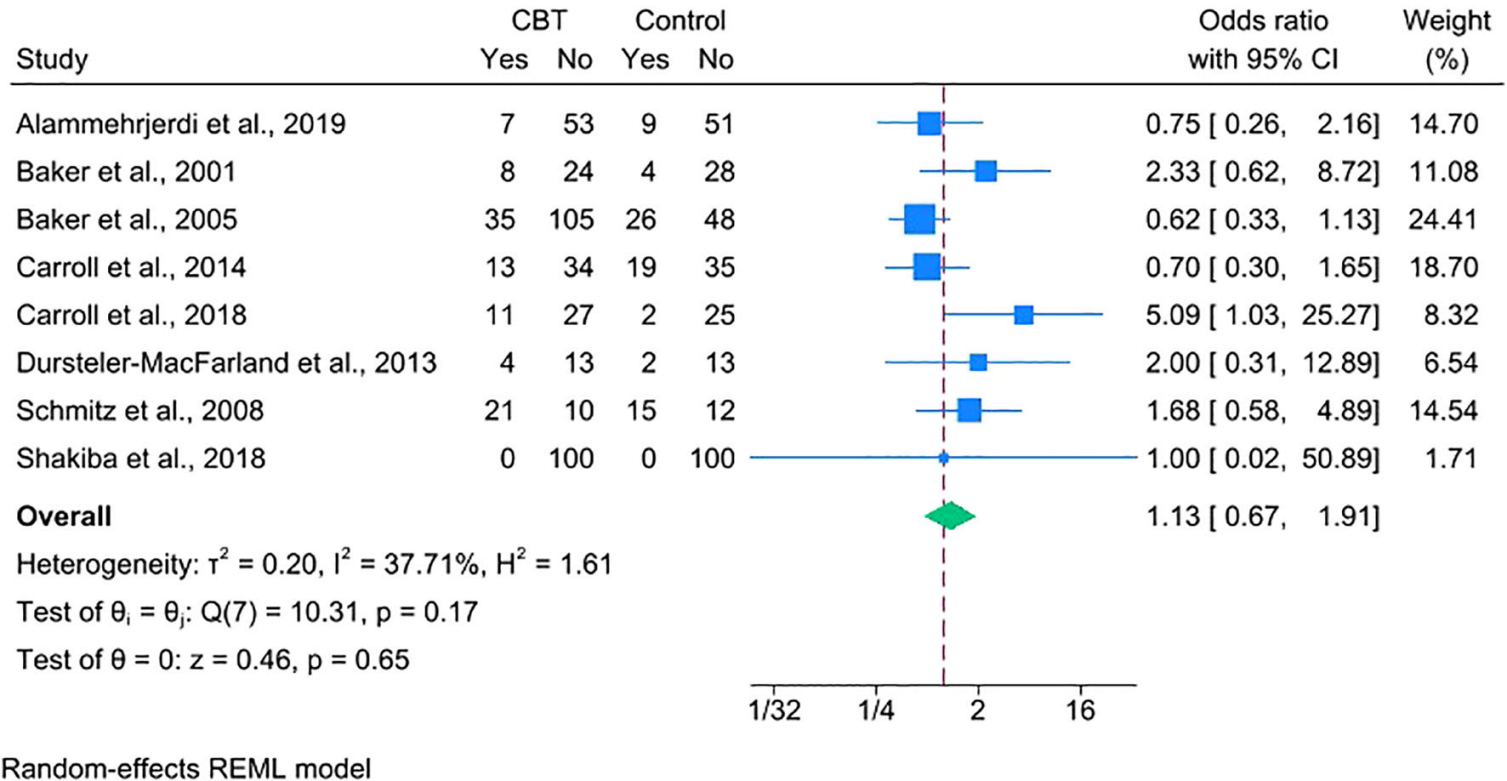
Forest plot comparing dropout rates between CBT and control group.

Treatment retention was assessed by comparing dropout rates across eight studies. The analysis showed no significant difference between the CBT and control groups (OR = 1.13, 95% CI = 0.67–1.91), indicating comparable retention (**Figure 4**). One trial was excluded from this analysis because it did not explicitly report dichotomous dropout data but had comparable treatment durations across arms (28).

The risk-of-bias assessment raised no serious concerns across the included studies (**Supplementary Figure S1**). However, a funnel plot analysis suggested potential publication bias, which was supported by a significant Egger’s test (*p* = 0.03) (**Supplementary Figures S2**, **S3**). Based on the GRADE framework, the certainty of the evidence for this outcome was rated as low, primarily due to serious imprecision and suspected publication bias (**Supplementary Table S1**).

Regarding safety, no trials reported adverse events attributable to the CBT intervention, supporting its tolerability. However, methods for monitoring these events varied. Several studies did not specify procedures for adverse event monitoring (22–24, 30). Others employed prospective surveillance, such as weekly checklists or safety monitoring boards (25–29).

### 3.6 Results of subgroup analysis

To investigate the sources of the substantial heterogeneity found in the primary analysis (I^2^ = 75.62%), we conducted five subgroup analyses based on prespecified and exploratory factors. The results are summarized in **Table 2**.

**Table 2:** Subgroup analyses for the primary outcome (stimulant abstinence).

Subgroup	No. of studies (No. of participants)	Odds ratio (95% CI)	I²	*P* for subgroup difference
Stimulant Type				0.05
Methamphetamine/Amphetamine	4 (598)	9.14 (1.69–49.35)	80.18	
Cocaine	4 (251)	1.26 (0.42–3.72)	57.47	
Geographic Region				< 0.001
Asia	2 (320)	70.44 (9.57–518.42)	0.00	
Non-Asia	6 (529)	2.22 (1.44–3.42)	0.00	
CBT Format				0.29
Individual	6 (757)	4.21 (1.03–17.11)	85.18	
Group	2 (92)	1.39 (0.32–6.02)	30.80	
Number of CBT Sessions				0.70
≤ 8 sessions	4 (499)	3.07 (1.88–5.02)	0.00	
> 8 sessions	4 (350)	1.93 (0.19–19.41)	82.97	
Outcome Assessment				0.87
Self-report	2 (278)	2.80 (1.55–5.04)	0.00	
Urine-verified	6 (571)	3.26 (0.59–18.08)	83.74	

The largest contrast was observed in the analysis by geographic region, which revealed a statistically significant difference between subgroups (*p* < 0.001). The pooled effect of CBT was markedly larger in studies conducted in Asia (2 studies (22, 30); OR = 70.44, 95% CI = 9.57–518.42) compared to those in non-Asian countries (6 studies (23–25, 27–29); OR = 2.22, 95% CI = 1.44–3.42). This stratification fully explained the between-study variance, with heterogeneity within both the Asian and non-Asian subgroups reduced to I^2^ = 0.00%.

A borderline significant difference was observed between subgroups based on stimulant type (*p* = 0.05). CBT demonstrated a significant effect for individuals with methamphetamine/amphetamine use disorders (4 studies (22–24, 30); OR = 9.14, 95% CI = 1.69–49.35), but a significant effect was not found for those with cocaine use disorders (4 studies (25, 27–29); OR = 1.26, 95% CI = 0.42–3.72). Substantial heterogeneity persisted within the methamphetamine/amphetamine subgroup (I^2^ = 80.18%).

The tests for subgroup differences were not statistically significant for CBT format (individual *vs*. group; *p* = 0.29), number of CBT sessions (≤ 8 *vs*. > 8; *p* = 0.70), or method of outcome assessment (self-report *vs*. urine-verified; *p* = 0.87).

### 3.7 Results of sensitivity analyses

To test the robustness of the primary findings, we performed several sensitivity analyses prespecified in the protocol.

**Figure 5:**
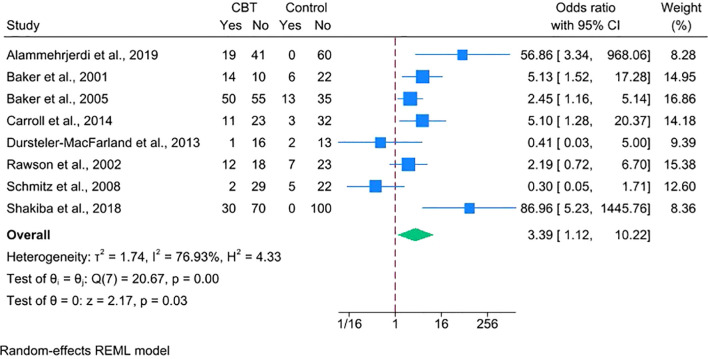
Forest plot depicting the efficacy of CBT on stimulant abstinence using per-protocol/completer data.

First, using per-protocol or completer datasets yielded a statistically significant benefit of CBT (OR = 3.39, 95% CI = 1.12–10.22) (**Figure 5**), consistent with the direction of the primary ITT estimate (OR = 2.88, 95% CI = 1.08–7.70). The wider CI reflects the loss of precision due to smaller analytic samples resulting from the exclusion of participants with missing endpoint data or protocol deviations.

**Figure 6:**
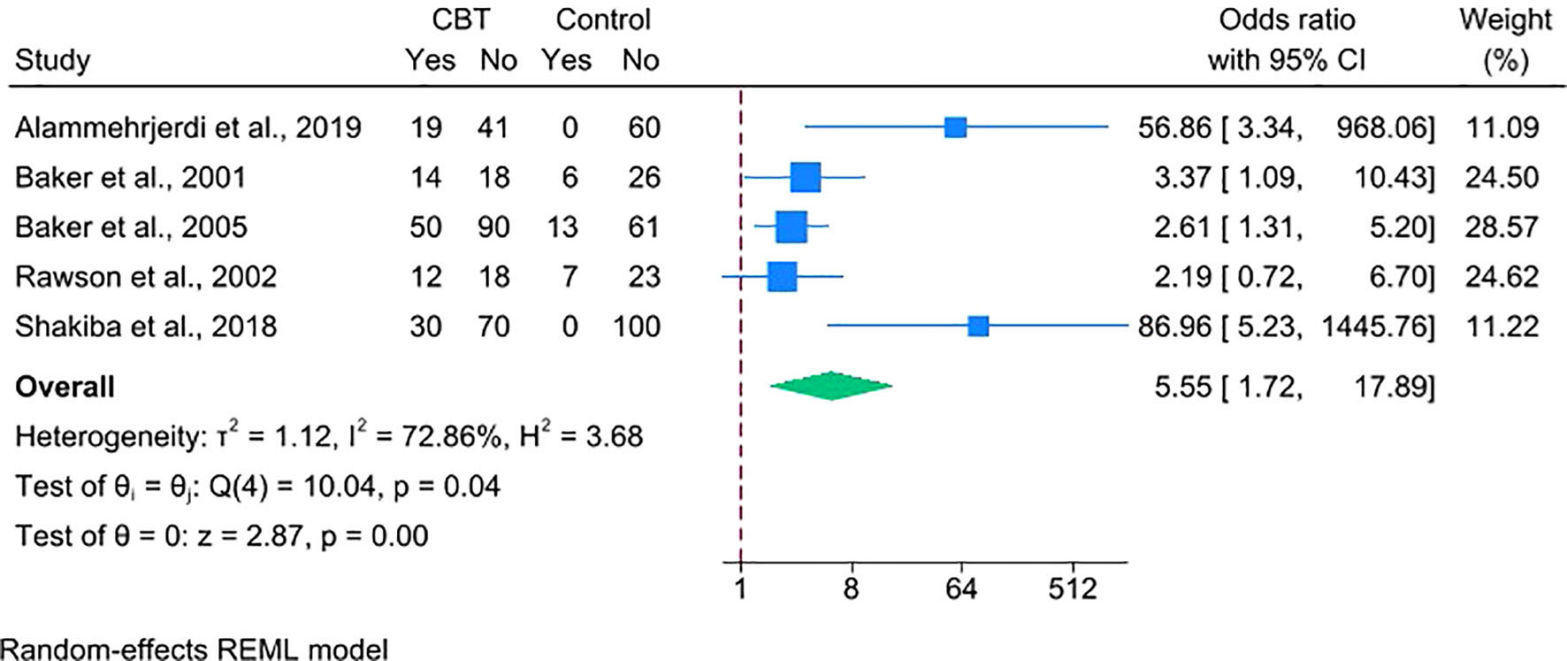
Forest plot depicting the efficacy of CBT on stimulant abstinence for point prevalence outcomes.

Second, restricting the analysis to the five trials (22–24, 28, 30) that assessed point-prevalence abstinence at treatment completion showed a larger effect (OR = 5.55, 95% CI = 1.72–17.89) (**Figure 6**), indicating that CBT’s benefit is especially evident at the end of treatment.

**Figure 7:**
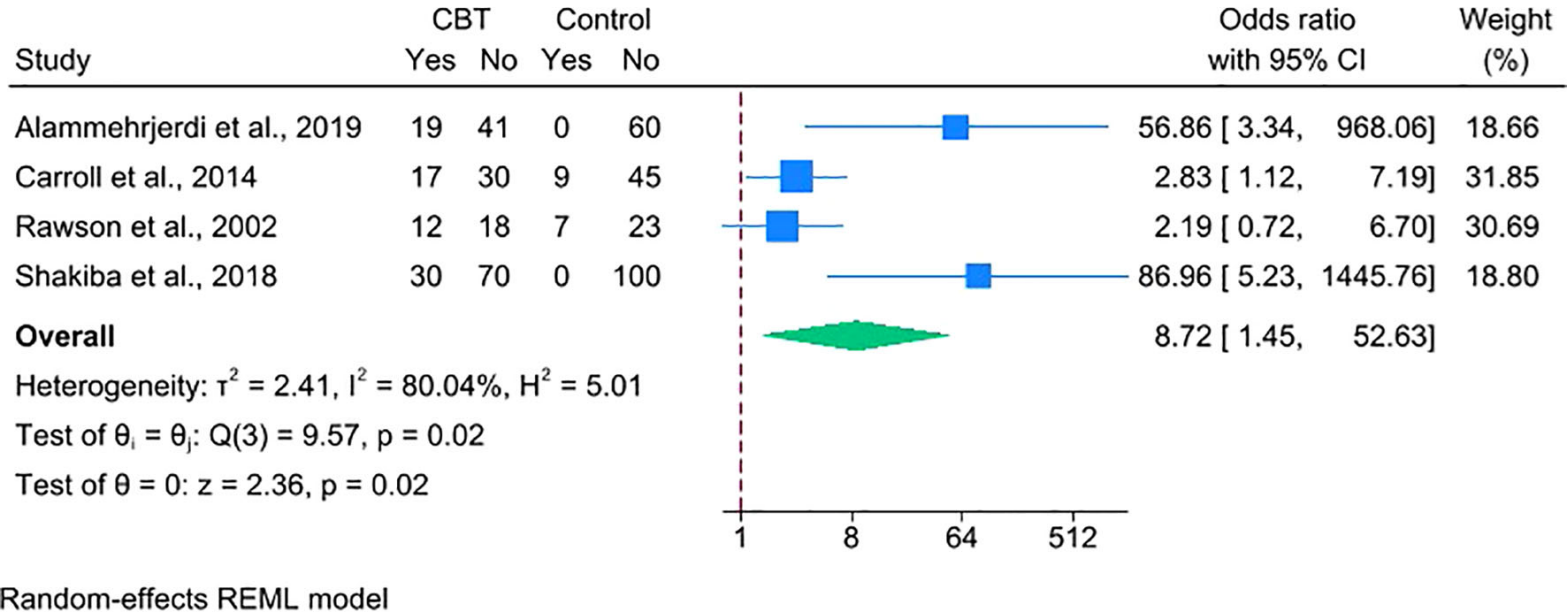
Forest plot depicting the efficacy of CBT on stimulant abstinence after excluding trials at overall high risk of bias.

Third, we conducted a RoB-informed analysis that excluded the four trials rated as having an overall high risk of bias. The remaining four comparisons (22, 25, 28, 30) continued to favor CBT (OR = 8.72, 95% CI 1.45–52.63; **Figure 7**). Heterogeneity persisted at a high level (I² = 80.04%), indicating that the variability was not explained by study-level risk of bias.

Because two trials (22, 30) in the evidence set had zero abstinence events in the control arm, we conducted a *post hoc* sparse-data sensitivity analysis excluding those studies to assess the influence of single-zero cells, which require continuity correction and can inflate study-level log ORs and between-study variance. In Shakiba et al. (30), the control-arm zero was assumed because outcome data were not reported, an assumption supported by universal baseline methamphetamine dependence and the authors’ statement that the control group showed no improvement. In Alammehrjerdi et al. (22), zero control-arm abstinence was explicitly reported. When both zero-control trials were removed, the pooled effect remained positive (OR = 2.22, 95% CI = 1.44–3.42) and residual heterogeneity disappeared (I² = 0.00%) (**Supplementary Figure S4**). This result aligns with the non-Asia stratum in the subgroup table (*k* = 6, OR = 2.22, 95% CI 1.44–3.42; I² = 0.00%), confirming that the apparent regional split serves as a proxy for the zero-event artifact rather than a clinical effect modifier.

Taken together, all sensitivity analyses support a beneficial effect of standalone CBT on short-term abstinence. The disappearance of heterogeneity after removing zero-control trials indicates that the between-study variability observed in the main ITT analysis (I² = 75.62%) reflects an explained methodological feature rather than true inconsistency across studies.

### 3.8 Publication bias assessment

Publication bias of the primary outcome was evaluated through visual inspection of the funnel plots and formal statistical testing. The standard funnel plot did not show marked asymmetry (**Supplementary Figure S5**). In the contour-enhanced funnel plot, studies were present in both non-significant (*p* > 0.10) and significant contours, rather than clustering only within statistically significant regions (**Supplementary Figure S6**). Consistent with these visual assessments, Egger’s test did not detect a significant small-study effect (β = 1.54, SE = 1.42, z = 1.09, *p* = 0.28).

However, this finding must be interpreted with considerable caution. It is well-established that with a small number of included studies (*k* = 8), both funnel plot inspection and statistical tests for publication bias, such as Egger’s test, have low statistical power to detect genuine bias reliably. The non-significant result should therefore not be viewed as conclusive evidence for the absence of publication bias.

Acknowledging this important limitation, while the possibility of underlying reporting biases cannot be definitively excluded, the analyses did not reveal a clear signal of asymmetry that would warrant a strong suspicion of publication bias. Therefore, based on the available evidence, publication bias was considered undetected in this analysis.

### 3.9 Long-term outcomes

Four trials (22, 25, 28, 30) provided follow-up data beyond the prespecified period (i.e., primary endpoint of each study or earliest follow-up). Carroll et al. (25) found that computerized CBT led to a continued monthly decline in cocaine use over six months, although no follow-up abstinence rates were reported. At 52 weeks, Rawson et al. (28) reported that CBT participants had higher rates of cocaine-negative urine samples (60% *vs*. 27%) and fewer self-reported use days compared to those in TAU. Two methamphetamine trials showed sustained effects at three months: Alammehrjerdi et al. (22) reported improvements in use frequency, days of use, and dependence severity, while Shakiba et al. (30) observed ongoing reductions in methamphetamine use based on the Opiate Treatment Index.

While these findings hint at the potential for durable benefits from CBT, no definitive conclusions about long-term efficacy can be drawn due to inconsistent follow-up durations and outcome measures across a limited number of studies.

### 3.10 Certainty of evidence

Based on the GRADE framework, the certainty of evidence for the effect of CBT on stimulant abstinence was rated as low (**Table 3**). This rating was influenced primarily by two concerns: a high risk of bias and serious imprecision.

**Table 3:** GRADE summary of findings: CBT vs. minimal-treatment controls for stimulant abstinence.

Certainty assessment							№ of patients		Effect		Certainty	Importance
№ of studies	Study design	Risk of bias	Inconsistency	Indirectness	Imprecision	Other considerations	CBT	Minimal-treatment controls	Relative (95% CI)	Absolute (95% CI)		
Stimulant Abstinence (follow-up: range 4 weeks to 24 weeks)												
8	randomized trials	serious^a^	not serious	not serious	serious^b^	publication bias undetected	145/457 (31.7%)	42/392 (10.7%)	OR 2.88 (1.08 to 7.70)	150 more per 1,000 (from 8 more to 373 more)	⊕⊕◯◯ Low	CRITICAL

a. The judgment for the Risk of bias domain was "serious," leading to a downgrade of one level. This decision was based on RoB2 assessments, which indicated that four of the eight RCTs included in the meta-analysis were rated as having a high overall risk of bias, primarily due to issues with missing outcome data (Domain 3) and the measurement of the outcome (Domain 4).

b. The judgment for the Imprecision domain was “serious,” leading to a downgrade of one level. The imprecision, reflected by a wide 95% CI (1.08–7.70), stems from two main issues: first, a small overall information base, and second, a statistical artifact caused by two trials with zero events in their control groups.

First, the evidence was downgraded one level because four of the eight included trials were judged to have a high overall risk of bias, mainly from missing outcome data and issues with outcome measurement. The downgrade was limited to one level because the direction of the effect remained consistent in sensitivity analyses that excluded these higher-risk studies. Second, the evidence was downgraded for serious imprecision. There were two core reasons for this judgment. The first was a small overall information base, with a limited number of trials and a modest number of events. The second was the statistical difficulty presented by two trials (22, 30) with zero abstinence events in their control groups. These factors, particularly the need for a continuity correction for the zero-event studies, increased the statistical variance and resulted in a wide and imprecise pooled CI (OR = 2.88, 95% CI = 1.08–7.70).

We did not downgrade for inconsistency because the substantial heterogeneity observed in the primary analysis (I² = 75.62%) was largely a statistical artifact attributable to the same two trials with zero events. When these trials were excluded in a *post hoc* sensitivity analysis, heterogeneity was eliminated (I² = 0.00%) while the positive treatment effect remained (OR = 2.22, 95% CI 1.44–3.42). This supports the interpretation that the variability was due to sparse data rather than true inconsistency in the treatment effect.

The evidence was not downgraded for indirectness, as the included studies aligned well with the review question, and publication bias was considered undetected, though the small number of studies limits this assessment.

## 4 Discussion

This systematic review and meta-analysis addressed a specific, practice-focused question: whether standalone CBT, delivered without additional active psychosocial interventions, improves abstinence in individuals with SUD compared with minimal-treatment controls. This question is of particular clinical relevance in settings where SUDs are rapidly emerging, yet the infrastructure to deliver resource-intensive specialized care (e.g., CM) is limited. The findings indicate that CBT alone increases short-term stimulant abstinence rates, and sensitivity analyses supported the stability of this effect. Furthermore, CBT demonstrated a retention rate comparable to minimal-treatment controls and was reported to be safe, with no adverse events noted in the included trials. According to the GRADE assessment, the certainty of the evidence was rated as low, warranting a cautious conclusion that CBT alone may offer a short−term abstinence benefit. Although the primary analysis focused on short-term outcomes, an exploratory review of long-term data across four RCTs suggested a consistent trend favoring CBT, though the variation in follow-up durations and outcome definitions precluded quantitative synthesis.

This signal, while uncertain, contrasts with several prior syntheses that found little or no effect of CBT. For example, the Cochrane review by Minozzi et al. (1) reported CBT *vs*. no intervention showed little to no difference in point abstinence at the end of treatment (RR = 1.05, 95% CI = 0.88–1.25), and De Crescenzo et al (9). found no clear advantage of CBT over TAU for end−of−treatment abstinence (OR = 1.17, 95% CI = 0.79–1.74). Reviews focused on amphetamine−type stimulants have also produced mixed results (10, 31). The likely reason for the discrepancy is methodological. We restricted eligibility to trials that compared standalone CBT with minimal-treatment controls; we excluded trials with combined interventions (e.g., Milby et al. (32), where CBT was paired with CM elements) and those with active comparators (e.g., Crits-Christoph et al. (33), in which the control arm of group drug counseling included a structured, manual-guided program based on the 12-step philosophy and peer support). This approach allowed us to evaluate the independent effect of CBT and avoid confounding influences that may have diluted effects in earlier reviews. As more psychosocial strategies accumulate evidence of benefit, the ethical equipoise for minimal-treatment control arms diminishes because withholding potentially effective care becomes increasingly difficult to justify. The resulting scarcity of such trials heightens the contribution of this meta-analysis, which compiles the available minimal-control comparisons to provide timely support for standalone CBT, particularly in settings where alternative resource-intensive evidence-based options are limited or infeasible.

Regarding treatment retention, CBT was found to be comparable to the control conditions. Across the trials that provided data, the odds of dropping out did not differ significantly between the CBT and control groups (OR = 1.13, 95% CI = 0.67–1.91). This contrasts with the Cochrane review of psychosocial interventions (1), which reported reduced dropout *vs*. no treatment (RR = 0.82, 95% CI = 0.74–0.91) and *vs*. TAU (RR = 0.79, 95% CI = 0.65–0.97). This discrepancy may be attributable to differences in the scope of the synthesized interventions. The Cochrane review included a broader set of interventions, including reinforcement-based strategies such as CM and CRA, whereas our analysis isolated standalone CBT without external reinforcement. In Cochrane’s subgroup analyses, CBT did not reduce dropout relative to no treatment (RR = 0.89, 95% CI = 0.68–1.17), which is consistent with our finding of comparable retention against minimal-treatment controls. Another factor that could have contributed to the discrepancy is the volume of evidence. The Cochrane review included a substantially larger number of trials overall, for instance, analyzing 30 studies with 4,078 participants for comparisons *vs*. no intervention. In contrast, our focused analysis was limited to eight RCTs with a total of 854 participants providing dropout data, which reduced statistical power to detect small differences.

The apparent regional differences revealed in the subgroup analysis are best interpreted as a methodological artifact rather than true effect modification. Both trials (22, 30) conducted in Asia contributed zero control-arm abstinence, which necessitated continuity corrections and increased between-study variance. When these sparse-data outliers were excluded, the pooled effect remained positive, and residual heterogeneity was eliminated. Given the very small number of Asian studies (*k* = 2) and the instability of their estimates, a true regional effect cannot be established. Instead, the findings point to an evidence gap and underscore the need for well-designed, transparently reported RCTs in Asian settings to obtain more reliable estimates in these regions where stimulant use is rapidly increasing. For instance, such trials should implement scheduled urine verification for all randomized participants. This avoids the risk of bias seen in Alammehrjerdi et al., where urine tests were performed only on participants who self-reported abstinence, potentially compromising the accuracy of the results.

Other exploratory subgroup analyses provided additional context. Although not statistically significant, there was a trend favoring individual over group CBT, which contrasts with previous research that found no difference in efficacy between the two formats (34). Shorter interventions, defined as eight sessions or fewer, consistently demonstrated a significant benefit. This suggests that less intensive CBT may be an efficient and practical option. This finding is consistent with previous dose–response studies that concluded shorter CBT schedules can be effective in reducing cocaine use (35). Analyses by stimulant type yielded borderline evidence of a difference (*p* = 0.05), suggesting potentially greater efficacy for methamphetamine/amphetamine than for cocaine use disorders. This divergence should be interpreted cautiously, as it may partly reflect a statistical artifact arising from the inclusion of two studies with zero control-arm events in the methamphetamine/amphetamine subgroup, which can inflate effect estimates. In addition to this methodological consideration, pharmacokinetic factors may also contribute. Cocaine’s shorter half-life compared to methamphetamine/amphetamine appears consistent with more rapid craving–use cycles (36–39), which could plausibly narrow the window for applying cognitive strategies during intense urges. In addition, some comparative evidence suggests that cocaine users tend to show greater deficits in verbal working memory compared to methamphetamine users (40), which might, in turn, make it more challenging to acquire and utilize CBT skills. Nevertheless, given the small evidence base and the borderline statistical significance, these explanations remain speculative. Additionally, no difference was detected by the outcome assessment method. Overall, the variability in CBT’s observed efficacy across studies seems to be explained more by a methodological issue stemming from specific trials with zero events in the control group, rather than by differences in the clinical characteristics of the intervention itself.

Our review’s strengths include a tightly framed question and a transparent, rigorous approach, yet important limitations remain. These limitations can be categorized into those related to our review process and those stemming from the evidence base itself. First, a limitation arises from our review process. Inclusion was restricted to peer-reviewed, English-language articles, which excluded grey literature and non-English studies and may have omitted relevant evidence. Second, further limitations stem from the evidence base included in the review. The certainty of the evidence for the primary outcome was low because several trials had a high risk of bias, and the overall sample size was small, leading to a wide CI around the pooled estimate. A sparse-data issue also contributed to this imprecision, as two trials had zero events in their control arms. Additionally, the reporting of longer-term outcomes was limited and heterogeneous in terms of timing and measures, precluding a robust synthesis of durability. Finally, the generalizability of the findings is limited. Most trials were conducted in Western settings, which restricts the applicability of our findings to the Asian context. This geographical bias is not unique to our selection of studies, as the Cochrane review (1) likewise indicates that trials evaluating CBT were conducted primarily in Western countries, underscoring this regional evidence gap.

Despite these limitations, the findings have practical and research implications. Although CM is supported by robust evidence, its implementation is often hindered by infrastructure, training, and funding needs (7). These challenges are particularly evident in resource-limited settings, such as the Republic of Korea and other parts of Asia (3, 5, 41). In Korea, CM is rarely used in practice. CBT, in contrast, is more readily scalable and can be delivered by trained clinicians within existing services without financial incentives. Its clinical relevance is further supported by neurobiological evidence suggesting that engagement with CBT may induce structural changes in brain regions involved in semantic processing and cognitive adaptation (42). On this basis, standalone CBT can be considered an early, deployable step within stepped−care pathways, especially where access to other resource-intensive evidence−based care is limited, provided its application is accompanied by rigorous outcome monitoring rather than assumed effectiveness.

To strengthen the evidence base, future research must address the methodological limitations identified in this review. Priority should be given to designing trials that successfully minimize participant attrition and employ robust outcome-assessment methods, such as blinded evaluations and biochemical verification of abstinence. Furthermore, there is a critical need for high-quality research in Asia. This review found only two regional trials, both with methodological limitations, and findings from Western settings cannot be generalized due to the influence of sociocultural context on psychotherapy. Therefore, well-designed RCTs in Asian settings are essential to address this evidence gap. Finally, to credibly estimate long-term effectiveness (i.e., the durability of treatment effects) and population-level impact, future trials should adopt standardized approaches to defining, measuring, and reporting long−term outcomes.

## 5 Conclusion

Compared with minimal-treatment controls, standalone CBT may increase short-term abstinence and shows similar retention, with no CBT-related adverse events reported. Because the GRADE assessment indicates low certainty, clinicians should use CBT cautiously and pair implementation with systematic outcome monitoring. Even so, its potential benefits, safety profile, and scalability relative to more resource-intensive options support continued use, particularly where intensive services are unavailable.

## Data Availability

The original contributions presented in the study are included in the article/**Supplementary Material**. Further inquiries can be directed to the corresponding author.
