# Supplementary material for: Efficacy of cognitive behavioral therapy for stimulant use disorders: a systematic review and meta-analysis
Source: Front Psychiatry. 2025 Nov 10;16:1695702. doi: 10.3389/fpsyt.2025.1695702 (PMC12640898; doi:10.3389/fpsyt.2025.1695702)
Supplement: Supplementary file 1 [file SupplementaryFile1.docx]

**Appendix A. Search strategies for each database**

**PubMed Search Strategy**

1. "Stimulant Use Disorder"[TW]
2. "Methamphetamine"[Mesh]
3. "Methamphetamine"[TW] OR "Deoxyephedrine"[TW] OR "Desoxyephedrine"[TW] OR "Methylamphetamine"[TW] OR "N-Methylamphetamine"[TW] OR "N Methylamphetamine"[TW] OR "Metamfetamine"[TW] OR "Methamphetamine Hydrochloride"[TW] OR "Hydrochloride, Methamphetamine"[TW] OR "Desoxyn"[TW] OR "Madrine"[TW]
4. "Amphetamine"[Mesh]
5. "Amphetamine"[TW] OR "Desoxynorephedrin"[TW] OR "Phenamine"[TW] OR "Phenopromin"[TW] OR "Amfetamine"[TW] OR "l-Amphetamine"[TW] OR "l Amphetamine"[TW] OR "Levoamphetamine"[TW] OR "levo-Amphetamine"[TW] OR "levo Amphetamine"[TW] OR "Amphetamine Sulfate"[TW] OR "Sulfate, Amphetamine"[TW] OR "Amphetamine Sulfate (2:1)"[TW] OR "Fenamine"[TW] OR "Mydrial"[TW] OR "Thyramine"[TW] OR "Centramina"[TW]
6. "Amphetamine-Related Disorders"[Mesh]
7. "Amphetamine-Related Disorders"[TW] OR "Amphetamine Related Disorders"[TW] OR "Disorder, Amphetamine-Related"[TW] OR "Disorders, Amphetamine-Related"[TW] OR "Amphetamine Abuse"[TW] OR "Abuse, Amphetamine"[TW] OR "Amphetamine Addiction"[TW] OR "Addiction, Amphetamine"[TW] OR "Amphetamine Dependence"[TW] OR "Dependence, Amphetamine"[TW]
8. "Cocaine"[Mesh]
9. "Cocaine"[TW] OR "Cocaine Hydrochloride"[TW] OR "Hydrochloride, Cocaine"[TW] OR "Cocaine HCl"[TW] OR "HCl, Cocaine"[TW]
10. "Cocaine-Related Disorders"[Mesh]
11. "Cocaine-Related Disorders"[TW] OR "Cocaine-Related Disorder"[TW] OR "Cocaine Related Disorders"[TW] OR "Disorder, Cocaine-Related"[TW] OR "Disorders, Cocaine-Related"[TW] OR "Cocaine Abuse"[TW] OR "Abuse, Cocaine"[TW] OR "Cocaine Dependence"[TW] OR "Dependence, Cocaine"[TW] OR "Dependences, Cocaine"[TW] OR "Cocaine Addiction"[TW] OR "Addiction, Cocaine"[TW]
12. 1 OR 2 OR 3 OR 4 OR 5 OR 6 OR 7 OR 8 OR 9 OR 10 OR 11
13. "Cognitive Behavioral Therapy"[Mesh]
14. "Cognitive Behavioral Therapy"[TW] OR "Behavioral Therapies, Cognitive"[TW] OR "Behavioral Therapy, Cognitive"[TW] OR "Cognitive Behavioral Therapies"[TW] OR "Therapies, Cognitive Behavioral"[TW] OR "Therapy, Cognitive Behavioral"[TW] OR "Cognition Therapy"[TW] OR "Cognition Therapies"[TW] OR "Therapies, Cognition"[TW] OR "Therapy, Cognitive Behavior"[TW] OR "Behavior Therapies, Cognitive"[TW] OR "Cognitive Behavior Therapies"[TW] OR "Therapies, Cognitive Behavior"[TW] OR "Therapy, Cognition"[TW] OR "Behavior Therapy, Cognitive"[TW] OR "Cognitive Behavior Therapy"[TW] OR "Cognitive Psychotherapy"[TW] OR "Cognitive Psychotherapies"[TW] OR "Psychotherapies, Cognitive"[TW] OR "Psychotherapy, Cognitive"[TW] OR "Therapy, Cognitive"[TW] OR "Cognitive Therapies"[TW] OR "Therapies, Cognitive"[TW] OR "Cognitive Behaviour Therapy"[TW] OR "Behaviour Therapies, Cognitive"[TW] OR "Behaviour Therapy, Cognitive"[TW] OR "Cognitive Behaviour Therapies"[TW] OR "Therapies, Cognitive Behaviour"[TW] OR "Therapy, Cognitive Behaviour"[TW] OR "Cognitive Therapy"[TW] OR "CBT"[TW] OR "cognitive-behavioural intervention"[TW] OR "cognitive-behavioural interventions"[TW] OR "cognitive-behavioral group therapy"[TW]
15. 13 OR 14
16. 12 AND 15
17. 16 AND ("Randomized Controlled Trial" [Publication Type] OR "Controlled Clinical Trial" [Publication Type] OR "Randomized Controlled Trials as Topic"[Mesh] OR "Random Allocation"[Mesh] OR "Double-Blind Method"[Mesh] OR "Single-Blind Method"[Mesh] OR "Clinical Trial" [Publication Type] OR "Clinical Trials as Topic"[Mesh] OR "Clinical Trial"[TW] OR ((singl*[TW] OR doubl*[TW] OR trebl*[TW] OR tripl*[TW]) AND (mask*[TW] OR blind*[TW])) OR "Placebos"[Mesh] OR placebo*[TW] OR random*[TW] OR "Research Design"[Mesh:NoExp]) NOT ("Animals"[Mesh] NOT "Humans"[Mesh])

**Embase Search Strategy**

1. "Stimulant Use Disorder":ti,ab,kw,de
2. "methamphetamine"/exp
3. "Methamphetamine":ti,ab,kw,de OR "Deoxyephedrine":ti,ab,kw,de OR "Desoxyephedrine":ti,ab,kw,de OR "Methylamphetamine":ti,ab,kw,de OR "N-Methylamphetamine":ti,ab,kw,de OR "N Methylamphetamine":ti,ab,kw,de OR "Metamfetamine":ti,ab,kw,de OR "Methamphetamine Hydrochloride":ti,ab,kw,de OR "Hydrochloride, Methamphetamine":ti,ab,kw,de OR "Desoxyn":ti,ab,kw,de OR "Madrine":ti,ab,kw,de
4. "amphetamine"/exp
5. "Amphetamine":ti,ab,kw,de OR "Desoxynorephedrin":ti,ab,kw,de OR "Phenamine":ti,ab,kw,de OR "Phenopromin":ti,ab,kw,de OR "Amfetamine":ti,ab,kw,de OR "l-Amphetamine":ti,ab,kw,de OR "l Amphetamine":ti,ab,kw,de OR "Levoamphetamine":ti,ab,kw,de OR "levo-Amphetamine":ti,ab,kw,de OR "levo Amphetamine":ti,ab,kw,de OR "Amphetamine Sulfate":ti,ab,kw,de OR "Sulfate, Amphetamine":ti,ab,kw,de OR "Amphetamine Sulfate (2:1)":ti,ab,kw,de OR "Fenamine":ti,ab,kw,de OR "Mydrial":ti,ab,kw,de OR "Thyramine":ti,ab,kw,de OR "Centramina":ti,ab,kw,de
6. "amphetamine dependence"/exp
7. "Amphetamine-Related Disorders":ti,ab,kw,de OR "Amphetamine Related Disorders":ti,ab,kw,de OR "Disorder, Amphetamine-Related":ti,ab,kw,de OR "Disorders, Amphetamine-Related":ti,ab,kw,de OR "Amphetamine Abuse":ti,ab,kw,de OR "Abuse, Amphetamine":ti,ab,kw,de OR "Amphetamine Addiction":ti,ab,kw,de OR "Addiction, Amphetamine":ti,ab,kw,de OR "Amphetamine Dependence":ti,ab,kw,de OR "Dependence, Amphetamine":ti,ab,kw,de
8. "cocaine"/exp
9. "Cocaine":ti,ab,kw,de OR "Cocaine Hydrochloride":ti,ab,kw,de OR "Hydrochloride, Cocaine":ti,ab,kw,de OR "Cocaine HCl":ti,ab,kw,de OR "HCl, Cocaine":ti,ab,kw,de
10. "cocaine-related disorder"/exp
11. "Cocaine-Related Disorders":ti,ab,kw,de OR "Cocaine-Related Disorder":ti,ab,kw,de OR "Cocaine Related Disorders":ti,ab,kw,de OR "Disorder, Cocaine-Related":ti,ab,kw,de OR "Disorders, Cocaine-Related":ti,ab,kw,de OR "Cocaine Abuse":ti,ab,kw,de OR "Abuse, Cocaine":ti,ab,kw,de OR "Cocaine Dependence":ti,ab,kw,de OR "Dependence, Cocaine":ti,ab,kw,de OR "Dependences, Cocaine":ti,ab,kw,de OR "Cocaine Addiction":ti,ab,kw,de OR "Addiction, Cocaine":ti,ab,kw,de
12. 1 OR 2 OR 3 OR 4 OR 5 OR 6 OR 7 OR 8 OR 9 OR 10 OR 11
13. "cognitive behavioral therapy"/exp
14. "Cognitive Behavioral Therapy":ti,ab,kw,de OR "Behavioral Therapies, Cognitive":ti,ab,kw,de OR "Behavioral Therapy, Cognitive":ti,ab,kw,de OR "Cognitive Behavioral Therapies":ti,ab,kw,de OR "Therapies, Cognitive Behavioral":ti,ab,kw,de OR "Therapy, Cognitive Behavioral":ti,ab,kw,de OR "Cognition Therapy":ti,ab,kw,de OR "Cognition Therapies":ti,ab,kw,de OR "Therapies, Cognition":ti,ab,kw,de OR "Therapy, Cognitive Behavior":ti,ab,kw,de OR "Behavior Therapies, Cognitive":ti,ab,kw,de OR "Cognitive Behavior Therapies":ti,ab,kw,de OR "Therapies, Cognitive Behavior":ti,ab,kw,de OR "Therapy, Cognition":ti,ab,kw,de OR "Behavior Therapy, Cognitive":ti,ab,kw,de OR "Cognitive Behavior Therapy":ti,ab,kw,de OR "Cognitive Psychotherapy":ti,ab,kw,de OR "Cognitive Psychotherapies":ti,ab,kw,de OR "Psychotherapies, Cognitive":ti,ab,kw,de OR "Psychotherapy, Cognitive":ti,ab,kw,de OR "Therapy, Cognitive":ti,ab,kw,de OR "Cognitive Therapies":ti,ab,kw,de OR "Therapies, Cognitive":ti,ab,kw,de OR "Cognitive Behaviour Therapy":ti,ab,kw,de OR "Behaviour Therapies, Cognitive":ti,ab,kw,de OR "Behaviour Therapy, Cognitive":ti,ab,kw,de OR "Cognitive Behaviour Therapies":ti,ab,kw,de OR "Therapies, Cognitive Behaviour":ti,ab,kw,de OR "Therapy, Cognitive Behaviour":ti,ab,kw,de OR "Cognitive Therapy":ti,ab,kw,de OR "CBT":ti,ab,kw,de OR "cognitive-behavioural intervention":ti,ab,kw,de OR "cognitive-behavioural interventions":ti,ab,kw,de OR "cognitive-behavioral group therapy":ti,ab,kw,de
15. 13 OR 14
16. 12 AND 15
17. 16 AND [randomized controlled trial]/lim

**PsycINFO Search Strategy**

1. tiab("Stimulant Use Disorder")
2. mainsubject("Methamphetamine")
3. tiab("Methamphetamine" OR "Deoxyephedrine" OR "Desoxyephedrine" OR "Methylamphetamine" OR "N-Methylamphetamine" OR "N Methylamphetamine" OR "Metamfetamine" OR "Methamphetamine Hydrochloride" OR "Hydrochloride, Methamphetamine" OR "Desoxyn" OR "Madrine")
4. mainsubject("Amphetamine")
5. tiab("Amphetamine" OR "Desoxynorephedrin" OR "Phenamine" OR "Phenopromin" OR "Amfetamine" OR "l-Amphetamine" OR "l Amphetamine" OR "Levoamphetamine" OR "levo-Amphetamine" OR "levo Amphetamine" OR "Amphetamine Sulfate" OR "Sulfate, Amphetamine" OR "Amphetamine Sulfate (2:1)" OR "Fenamine" OR "Mydrial" OR "Thyramine" OR "Centramina")
6. mainsubject("Amphetamine-Related Disorders")
7. tiab("Amphetamine-Related Disorders" OR "Amphetamine Related Disorders" OR "Disorder, Amphetamine-Related" OR "Disorders, Amphetamine-Related" OR "Amphetamine Abuse" OR "Abuse, Amphetamine" OR "Amphetamine Addiction" OR "Addiction, Amphetamine" OR "Amphetamine Dependence" OR "Dependence, Amphetamine")
8. mainsubject("Cocaine")
9. tiab("Cocaine" OR "Cocaine Hydrochloride" OR "Hydrochloride, Cocaine" OR "Cocaine HCl" OR "HCl, Cocaine")
10. mainsubject("Cocaine-Related Disorders")
11. tiab("Cocaine-Related Disorders" OR "Cocaine-Related Disorder" OR "Cocaine Related Disorders" OR "Disorder, Cocaine-Related" OR "Disorders, Cocaine-Related" OR "Cocaine Abuse" OR "Abuse, Cocaine" OR "Cocaine Dependence" OR "Dependence, Cocaine" OR "Dependences, Cocaine" OR "Cocaine Addiction" OR "Addiction, Cocaine")
12. 1 OR 2 OR 3 OR 4 OR 5 OR 6 OR 7 OR 8 OR 9 OR 10 OR 11
13. mainsubject("Cognitive Behavioral Therapy")
14. tiab("Cognitive Behavioral Therapy" OR "Behavioral Therapies, Cognitive" OR "Behavioral Therapy, Cognitive" OR "Cognitive Behavioral Therapies" OR "Therapies, Cognitive Behavioral" OR "Therapy, Cognitive Behavioral" OR "Cognition Therapy" OR "Cognition Therapies" OR "Therapies, Cognition" OR "Therapy, Cognitive Behavior" OR "Behavior Therapies, Cognitive" OR "Cognitive Behavior Therapies" OR "Therapies, Cognitive Behavior" OR "Therapy, Cognition" OR "Behavior Therapy, Cognitive" OR "Cognitive Behavior Therapy" OR "Cognitive Psychotherapy" OR "Cognitive Psychotherapies" OR "Psychotherapies, Cognitive" OR "Psychotherapy, Cognitive" OR "Therapy, Cognitive" OR "Cognitive Therapies" OR "Therapies, Cognitive" OR "Cognitive Behaviour Therapy" OR "Behaviour Therapies, Cognitive" OR "Behaviour Therapy, Cognitive" OR "Cognitive Behaviour Therapies" OR "Therapies, Cognitive Behaviour" OR "Therapy, Cognitive Behaviour" OR "Cognitive Therapy" OR "CBT" OR "cognitive-behavioural intervention" OR "cognitive-behavioural interventions" OR "cognitive-behavioral group therapy")
15. 13 OR 14
16. 12 AND 15

**Cochrane Library Search Strategy**

1. "Stimulant Use Disorder":ti,ab,kw
2. [mh "Methamphetamine"]
3. "Methamphetamine":ti,ab,kw OR "Deoxyephedrine":ti,ab,kw OR "Desoxyephedrine":ti,ab,kw OR "Methylamphetamine":ti,ab,kw OR "N-Methylamphetamine":ti,ab,kw OR "N Methylamphetamine":ti,ab,kw OR "Metamfetamine":ti,ab,kw OR "Methamphetamine Hydrochloride":ti,ab,kw OR "Hydrochloride, Methamphetamine":ti,ab,kw OR "Desoxyn":ti,ab,kw OR "Madrine":ti,ab,kw
4. [mh "Amphetamine"]
5. "Amphetamine":ti,ab,kw OR "Desoxynorephedrin":ti,ab,kw OR "Phenamine":ti,ab,kw OR "Phenopromin":ti,ab,kw OR "Amfetamine":ti,ab,kw OR "l-Amphetamine":ti,ab,kw OR "l Amphetamine":ti,ab,kw OR "Levoamphetamine":ti,ab,kw OR "levo-Amphetamine":ti,ab,kw OR "levo Amphetamine":ti,ab,kw OR "Amphetamine Sulfate":ti,ab,kw OR "Sulfate, Amphetamine":ti,ab,kw OR "Amphetamine Sulfate (2:1)":ti,ab,kw OR "Fenamine":ti,ab,kw OR "Mydrial":ti,ab,kw OR "Thyramine":ti,ab,kw OR "Centramina":ti,ab,kw
6. [mh "Amphetamine-Related Disorders"]
7. "Amphetamine-Related Disorders":ti,ab,kw OR "Amphetamine Related Disorders":ti,ab,kw OR "Disorder, Amphetamine-Related":ti,ab,kw OR "Disorders, Amphetamine-Related":ti,ab,kw OR "Amphetamine Abuse":ti,ab,kw OR "Abuse, Amphetamine":ti,ab,kw OR "Amphetamine Addiction":ti,ab,kw OR "Addiction, Amphetamine":ti,ab,kw OR "Amphetamine Dependence":ti,ab,kw OR "Dependence, Amphetamine":ti,ab,kw
8. [mh "Cocaine"]
9. "Cocaine":ti,ab,kw OR "Cocaine Hydrochloride":ti,ab,kw OR "Hydrochloride, Cocaine":ti,ab,kw OR "Cocaine HCl":ti,ab,kw OR "HCl, Cocaine":ti,ab,kw
10. [mh "Cocaine-Related Disorders"]
11. "Cocaine-Related Disorders":ti,ab,kw OR "Cocaine-Related Disorder":ti,ab,kw OR "Cocaine Related Disorders":ti,ab,kw OR "Disorder, Cocaine-Related":ti,ab,kw OR "Disorders, Cocaine-Related":ti,ab,kw OR "Cocaine Abuse":ti,ab,kw OR "Abuse, Cocaine":ti,ab,kw OR "Cocaine Dependence":ti,ab,kw OR "Dependence, Cocaine":ti,ab,kw OR "Dependences, Cocaine":ti,ab,kw OR "Cocaine Addiction":ti,ab,kw OR "Addiction, Cocaine":ti,ab,kw
12. 1 OR 2 OR 3 OR 4 OR 5 OR 6 OR 7 OR 8 OR 9 OR 10 OR 11
13. [mh "Cognitive Behavioral Therapy"]
14. "Cognitive Behavioral Therapy":ti,ab,kw OR "Behavioral Therapies, Cognitive":ti,ab,kw OR "Behavioral Therapy, Cognitive":ti,ab,kw OR "Cognitive Behavioral Therapies":ti,ab,kw OR "Therapies, Cognitive Behavioral":ti,ab,kw OR "Therapy, Cognitive Behavioral":ti,ab,kw OR "Cognition Therapy":ti,ab,kw OR "Cognition Therapies":ti,ab,kw OR "Therapies, Cognition":ti,ab,kw OR "Therapy, Cognitive Behavior":ti,ab,kw OR "Behavior Therapies, Cognitive":ti,ab,kw OR "Cognitive Behavior Therapies":ti,ab,kw OR "Therapies, Cognitive Behavior":ti,ab,kw OR "Therapy, Cognition":ti,ab,kw OR "Behavior Therapy, Cognitive":ti,ab,kw OR "Cognitive Behavior Therapy":ti,ab,kw OR "Cognitive Psychotherapy":ti,ab,kw OR "Cognitive Psychotherapies":ti,ab,kw OR "Psychotherapies, Cognitive":ti,ab,kw OR "Psychotherapy, Cognitive":ti,ab,kw OR "Therapy, Cognitive":ti,ab,kw OR "Cognitive Therapies":ti,ab,kw OR "Therapies, Cognitive":ti,ab,kw OR "Cognitive Behaviour Therapy":ti,ab,kw OR "Behaviour Therapies, Cognitive":ti,ab,kw OR "Behaviour Therapy, Cognitive":ti,ab,kw OR "Cognitive Behaviour Therapies":ti,ab,kw OR "Therapies, Cognitive Behaviour":ti,ab,kw OR "Therapy, Cognitive Behaviour":ti,ab,kw OR "Cognitive Therapy":ti,ab,kw OR "CBT":ti,ab,kw OR "cognitive-behavioural intervention":ti,ab,kw OR "cognitive-behavioural interventions":ti,ab,kw OR "cognitive-behavioral group therapy":ti,ab,kw
15. 13 OR 14
16. 12 AND 15

**Appendix B. Studies excluded at full-text stage with reasons**

**Integrating other interventions (n=12)**

1. Higgins ST, Budney AJ, Bickel WK, Hughes JR, Foerg F, Badger G. Achieving cocaine abstinence with a behavioral approach. Am J Psychiatry (1993) 150(5):763–9. doi: 10.1176/ajp.150.5.763
2. Higgins ST, Sigmon SC, Wong CJ, Heil SH, Badger GJ, Donham RL, et al. Community reinforcement therapy for cocaine‑dependent outpatients. Arch Gen Psychiatry (2003) 60(10):1043–52. doi: 10.1001/archpsyc.60.9.1043
3. Magura S, Rosenblum A, Fong C, Villano C, Richman B. Treating cocaine‑using methadone patients: predictors of outcomes in a psychosocial clinical trial. Subst Use Misuse (2002) 37(14):1927–55. doi: 10.1081/ja-120016225
4. McKee SA, Carroll KM, Sinha R, Robinson JE, Nich C, Cavallo D, et al. Enhancing brief cognitive‑behavioral therapy with motivational enhancement techniques in cocaine users. Drug Alcohol Depend (2007) 91(1):97–101. doi: 10.1016/j.drugalcdep.2007.05.006
5. Milby JB, Schumacher JE, Vuchinich RE, Freedman MJ, Kertesz S, Wallace D. Toward cost‑effective initial care for substance‑abusing homeless. J Subst Abuse Treat (2008) 34(2):180–91. doi: 10.1016/j.jsat.2007.03.003
6. Mimiaga MJ, Pantalone DW, Biello KB, Hughto JMW, Frank J, O’Cleirigh C, et al. An initial randomized controlled trial of behavioral activation for treatment of concurrent crystal methamphetamine dependence and sexual risk for HIV acquisition among men who have sex with men. AIDS Care (2019) 31(9):1083–95. doi: 10.1080/09540121.2019.1595518
7. Parsons JT, John SA, Millar BM, Starks TJ. Testing the efficacy of combined motivational interviewing and cognitive behavioral skills training to reduce methamphetamine use and improve HIV medication adherence among HIV‑positive gay and bisexual men. AIDS Behav (2018) 22(8):2674–86. doi: 10.1007/s10461-018-2086-5
8. Pitpitan EV, Semple SJ, Zians J, Strathdee SA, Patterson TL. Mood, meth, condom use, and gender: latent growth curve modeling results from a randomized trial. AIDS Behav (2018) 22(9):2815–29. doi: 10.1007/s10461-018-2147-9
9. Rowan‑Szal GA, Bartholomew NG, Chatham LR, Simpson DD. A combined cognitive and behavioral intervention for cocaine‑using methadone clients. J Psychoactive Drugs (2005) 37(1):75–84. doi: 10.1080/02791072.2005.10399750
10. Schaub MP, Castro RP, Wenger A, Baumgartner C, Stark L, Ebert DD, et al. Web‑based self‑help with and without chat counseling to reduce cocaine use in cocaine misusers: results of a three‑arm randomized controlled trial. Internet Interv (2019) 17:100251. doi: 10.1016/j.invent.2019.100251
11. Tait RJ, McKetin R, Kay‑Lambkin F, Carron‑Arthur B, Bennett A, Bennett K, et al. A web‑based intervention for users of amphetamine‑type stimulants: 3‑month outcomes of a randomized controlled trial. JMIR Ment Health (2014) 1(1):e1. doi: 10.2196/mental.3278
12. Tait RJ, McKetin R, Kay‑Lambkin F, Carron‑Arthur B, Bennett A, Bennett K, et al. Six‑month outcomes of a web‑based intervention for users of amphetamine‑type stimulants: randomized controlled trial. J Med Internet Res (2015) 17(4):e105. doi: 10.2196/jmir.3778

**Non-CBT intervention (n=2)**

1. Martin G, Copeland J. Brief intervention for regular ecstasy (MDMA) users: pilot randomized trial of a check‑up model. J Subst Use (2010) 15(2):131–42. doi: 10.3109/14659890903075074
2. Srisurapanont M, Sombatmai S, Boripuntakul T. Brief intervention for students with methamphetamine use disorders: a randomized controlled trial. Am J Addict (2007) 16(2):111–16. doi: 10.1080/10550490601184431

**CBT as aftercare (n=2)**

1. McKay JR, Alterman AI, Cacciola JS, Rutherford MJ, O’Brien CP, Koppenhaver J. Group counseling versus individualized relapse prevention aftercare following intensive outpatient treatment for cocaine dependence: initial results. J Consult Clin Psychol (1997) 65(5):778–88. doi: 10.1037/0022-006x.65.5.778
2. McKay JR, Lynch KG, Coviello D, Morrison R, Cary MS, Skalina L, et al. Randomized trial of continuing care enhancements for cocaine‑dependent patients following initial engagement. J Consult Clin Psychol (2010) 78(1):111–20. doi: 10.1037/a0018139

**CBT not solely targeting stimulant use reduction (n=1)**

1. Glasner S, Patrick K, Ybarra M, Reback CJ, Ang A, Kalichman S, et al. Promising outcomes from a cognitive behavioral therapy text‑messaging intervention targeting drug use, antiretroviral therapy adherence, and HIV risk behaviors among adults living with HIV and substance use disorders. Drug Alcohol Depend (2022) 231:109229. doi: 10.1016/j.drugalcdep.2021.109229

**Comparator with active component (n=13)**

1. Carroll KM, Rounsaville BJ, Gordon LT, Nich C, Jatlow P, Bisighini RM, et al. Psychotherapy and pharmacotherapy for ambulatory cocaine abusers. Arch Gen Psychiatry (1994) 51(3):177–87. doi: 10.1001/archpsyc.1994.03950030013002
2. Carroll KM, Nich C, Ball SA, McCance E, Rounsaville BJ. Treatment of cocaine and alcohol dependence with psychotherapy and disulfiram. Addiction (1998) 93(5):713–27. doi: 10.1046/j.1360-0443.1998.9357137.x
3. Crits‑Christoph P, Siqueland L, Blaine J, Frank A, Luborsky L, Onken LS, et al. Psychosocial treatments for cocaine dependence: National Institute on Drug Abuse collaborative cocaine treatment study. Arch Gen Psychiatry (1999) 56(6):493–502. doi: 10.1001/archpsyc.56.6.493
4. Darker CD, Sweeney B, El Hassan H, Kelly A, Smyth BP, Barry J. Cognitive behavioural coping skills therapy in cocaine using methadone maintained patients: a pilot randomised controlled trial. Heroin Addict Relat Clin Probl (2012) 14(3):101–10.
5. Epstein DH, Hawkins WE, Covi L, Umbricht A, Preston KL. Cognitive‑behavioral therapy plus contingency management for cocaine use: findings during treatment and across 12‑month follow‑up. Psychol Addict Behav (2003) 17(1):73–82. doi: 10.1037/0893-164x.17.1.73
6. Harada T, Shirasaka T, Baba T, Mizusawa A, Villaroman A, Noguera‑Caoile R, et al. Residential cognitive‑behavioral therapy versus therapeutic community for patients with methamphetamine use disorders in the Philippines: a randomized controlled trial. Addict Behav Rep (2025) 21:100605. doi: 10.1016/j.abrep.2025.100605
7. Hoffman JA, Caudill BD, Koman JJ 3rd, Luckey JW, Flynn PM, Mayo DW. Psychosocial treatments for cocaine abuse: 12‑month treatment outcomes. J Subst Abuse Treat (1996) 13(1):3–11. doi: 10.1016/0740-5472(95)02020-9
8. Marsden J, Goetz C, Meynen T, Mitcheson L, Stillwell G, Eastwood B, et al. Memory‑focused cognitive therapy for cocaine use disorder: theory, procedures and preliminary evidence from an external pilot randomised controlled trial. EBioMedicine (2018) 29:177–89. doi: 10.1016/j.ebiom.2018.01.039
9. McKay JR, van Horn D, Ivey M, Drapkin ML, Rennert L, Lynch KG. Enhanced continuing care provided in parallel to intensive outpatient treatment does not improve outcomes for patients with cocaine dependence. J Stud Alcohol Drugs (2013) 74(4):642–51. doi: 10.15288/jsad.2013.74.642
10. Secades‑Villa R, Sánchez‑Hervás E, Zacarés‑Romaguera F, García‑Rodríguez O, Santonja‑Gómez FJ, García‑Fernández G. Community Reinforcement Approach (CRA) for cocaine dependence in the Spanish public health system: 1‑year outcome. Drug Alcohol Rev (2011) 30(6):606–12. doi: 10.1111/j.1465-3362.2010.00250.x
11. Shoptaw S, Reback CJ, Larkins S, Wang PC, Rotheram‑Fuller E, Dang J, et al. Outcomes using two tailored behavioral treatments for substance abuse in urban gay and bisexual men. J Subst Abuse Treat (2008) 35(3):285–93. doi: 10.1016/j.jsat.2007.11.004
12. Takano A, Miyamoto Y, Shinozaki T, Matsumoto T, Kawakami N. Effect of a web‑based relapse prevention program on abstinence among Japanese drug users: a pilot randomized controlled trial. J Subst Abuse Treat (2020) 111:37–46. doi: 10.1016/j.jsat.2019.12.001
13. Woody GE, Gallop R, Luborsky L, Blaine J, Frank A, Salloum IM, et al. HIV risk reduction in the National Institute on Drug Abuse cocaine collaborative treatment study. J Acquir Immune Defic Syndr (2003) 33(1):82–87. doi: 10.1097/00126334-200305010-00012

**Appendix C. Risk of bias assessment for the secondary outcome of treatment dropout**


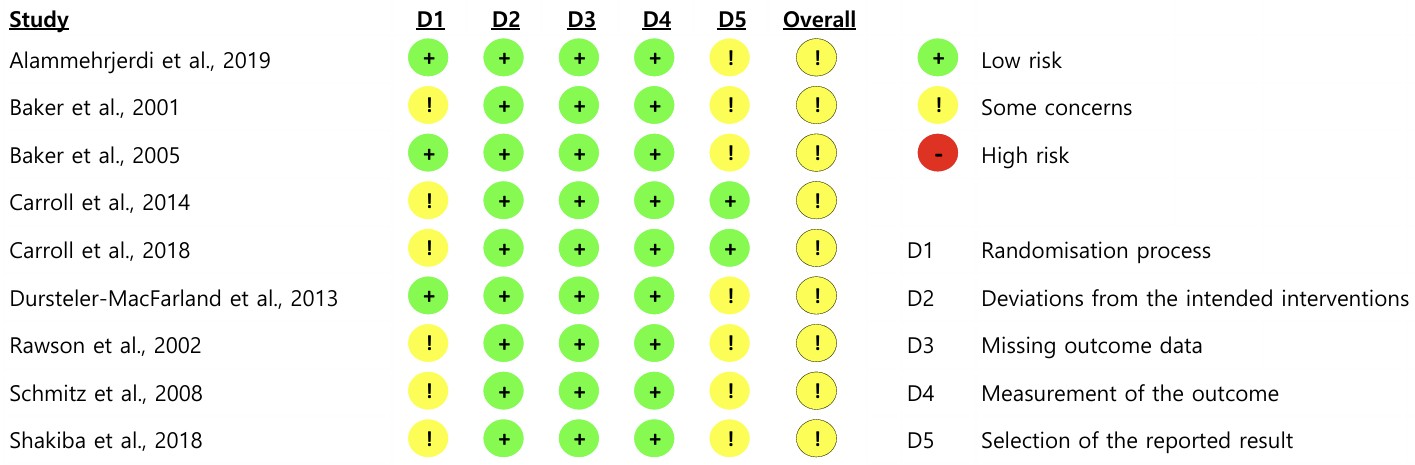


Supplementary Figure S1. Risk of bias assessment for the secondary outcome of treatment dropout

**Appendix D. Funnel plots for publication bias assessment for the secondary outcome of treatment dropout**

**
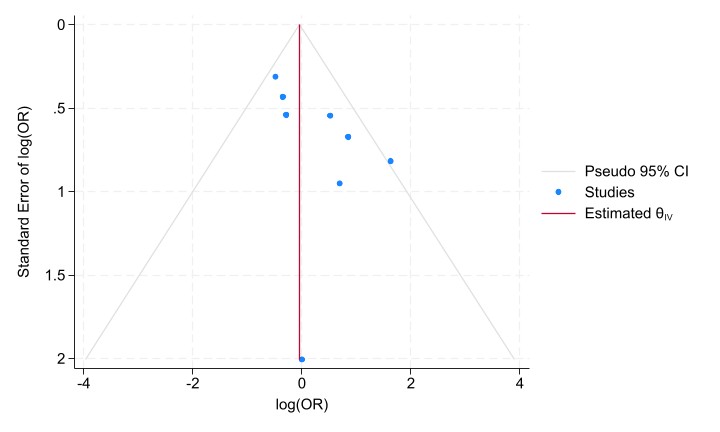
**

Supplementary Figure S2. Funnel plot for the secondary outcome of treatment dropout

**
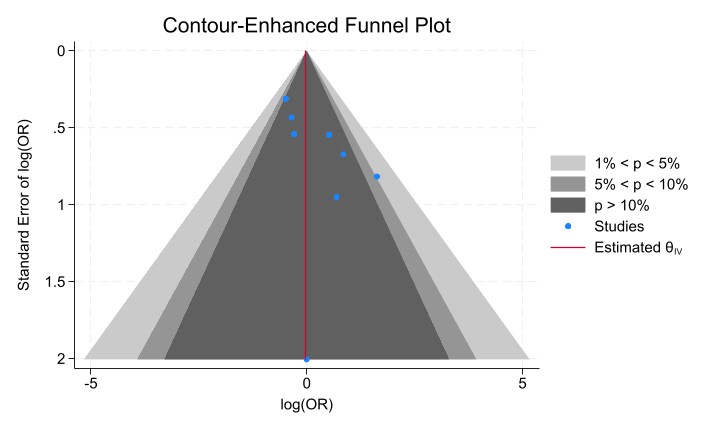
**

Supplementary Figure S3. Contour-enhanced funnel plot for the secondary outcome of treatment dropout

**Appendix E. GRADE Summary of Findings for the secondary outcome of treatment dropout**

Supplementary Table S1. GRADE Summary of Findings: CBT vs. minimal-treatment controls for treatment dropout

| **Certainty assessment** | | | | | | | **№ of patients** | | **Effect** | | **Certainty** | **Importance** |
| --- | --- | --- | --- | --- | --- | --- | --- | --- | --- | --- | --- | --- |
| **№ of studies** | **Study design** | **Risk of bias** | **Inconsistency** | **Indirectness** | **Imprecision** | **Other considerations** | **CBT** | **Minimal-treatment controls** | **Relative (95% CI)** | **Absolute (95% CI)** |  |  |
| **Dropout (follow-up: range 4 weeks to 24 weeks)** | | | | | | | | | | | | |
| 8 | randomized trials | not serious | not serious | not serious | serious^a^ | publication bias suspected^b^ | 99/465 (21.3%) | 77/389 (19.8%) | **OR 1.13** (0.67–1.91) | **20 more per 1,000** (from 56 fewer to 122 more) | ⨁⨁◯◯ Low | CRITICAL |

1. Downgraded one level for the Imprecision domain, as indicated by a wide 95% CI (0.67–1.91) that crosses the line of no effect and a small overall information base.
2. Downgraded one level for suspected publication bias, as indicated by visual asymmetry in the funnel plot and a statistically significant Egger’s test (*p* = 0.03). Caution is warranted in this interpretation due to the small number of studies (*k* < 10).

**Appendix F. Efficacy of CBT on stimulant abstinence from a post-hoc sensitivity analysis excluding studies with zero control group events**


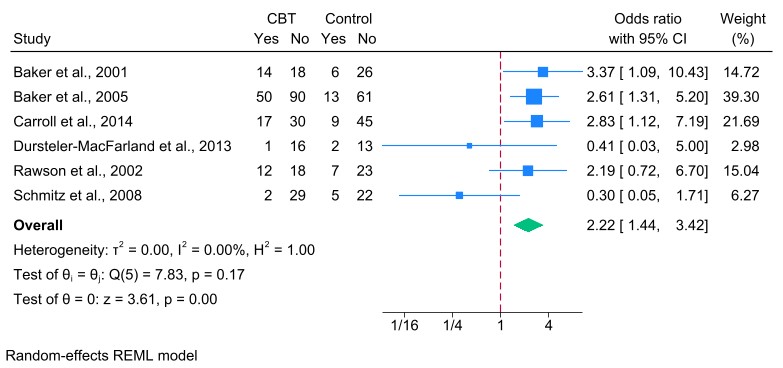


Supplementary Figure S4. Forest plot of post-hoc sensitivity analysis for the efficacy of CBT on stimulant abstinence

**Appendix G. Funnel plots for publication bias assessment for the primary outcome of stimulant abstinence**

**
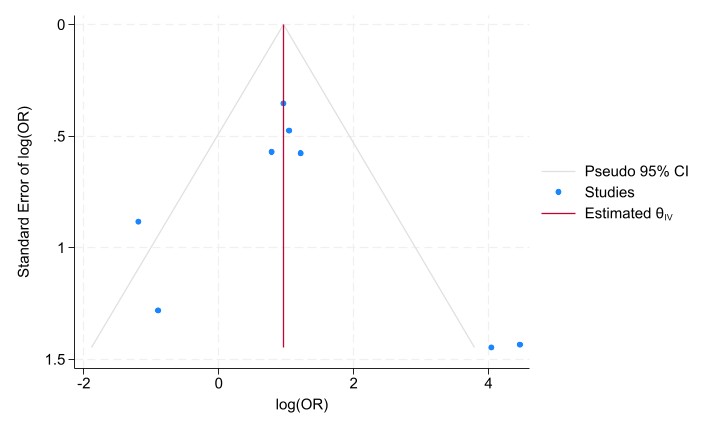
**

Supplementary Figure S5. Funnel plot for the primary outcome of stimulant abstinence

**
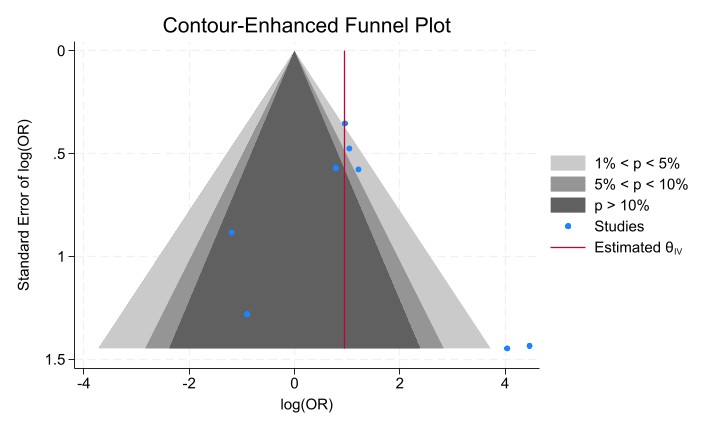
**

Supplementary Figure S6. Contour-enhanced funnel plot for the primary outcome of stimulant abstinence

**Appendix H. PRISMA 2020 Checklist**

| **Section and Topic** | **Item #** | **Checklist item** | **Location where item is reported** |
| --- | --- | --- | --- |
| **TITLE** | | |  |
| Title | 1 | Identify the report as a systematic review. | 1 |
| **ABSTRACT** | | |  |
| Abstract | 2 | See the PRISMA 2020 for Abstracts checklist. | 2 |
| **INTRODUCTION** | | |  |
| Rationale | 3 | Describe the rationale for the review in the context of existing knowledge. | 3 |
| Objectives | 4 | Provide an explicit statement of the objective(s) or question(s) the review addresses. | 3 |
| **METHODS** | | |  |
| Eligibility criteria | 5 | Specify the inclusion and exclusion criteria for the review and how studies were grouped for the syntheses. | 4-5 |
| Information sources | 6 | Specify all databases, registers, websites, organisations, reference lists and other sources searched or consulted to identify studies. Specify the date when each source was last searched or consulted. | 5 |
| Search strategy | 7 | Present the full search strategies for all databases, registers and websites, including any filters and limits used. | 5 |
| Selection process | 8 | Specify the methods used to decide whether a study met the inclusion criteria of the review, including how many reviewers screened each record and each report retrieved, whether they worked independently, and if applicable, details of automation tools used in the process. | 5 |
| Data collection process | 9 | Specify the methods used to collect data from reports, including how many reviewers collected data from each report, whether they worked independently, any processes for obtaining or confirming data from study investigators, and if applicable, details of automation tools used in the process. | 5 |
| Data items | 10a | List and define all outcomes for which data were sought. Specify whether all results that were compatible with each outcome domain in each study were sought (e.g. for all measures, time points, analyses), and if not, the methods used to decide which results to collect. | 5 |
|  | 10b | List and define all other variables for which data were sought (e.g. participant and intervention characteristics, funding sources). Describe any assumptions made about any missing or unclear information. | 5 |
| Study risk of bias assessment | 11 | Specify the methods used to assess risk of bias in the included studies, including details of the tool(s) used, how many reviewers assessed each study and whether they worked independently, and if applicable, details of automation tools used in the process. | 5-6 |
| Effect measures | 12 | Specify for each outcome the effect measure(s) (e.g. risk ratio, mean difference) used in the synthesis or presentation of results. | 6 |
| Synthesis methods | 13a | Describe the processes used to decide which studies were eligible for each synthesis (e.g. tabulating the study intervention characteristics and comparing against the planned groups for each synthesis (item #5)). | 6 |
|  | 13b | Describe any methods required to prepare the data for presentation or synthesis, such as handling of missing summary statistics, or data conversions. | 6 |
|  | 13c | Describe any methods used to tabulate or visually display results of individual studies and syntheses. | 6 |
|  | 13d | Describe any methods used to synthesize results and provide a rationale for the choice(s). If meta-analysis was performed, describe the model(s), method(s) to identify the presence and extent of statistical heterogeneity, and software package(s) used. | 6 |
|  | 13e | Describe any methods used to explore possible causes of heterogeneity among study results (e.g. subgroup analysis, meta-regression). | 6 |
|  | 13f | Describe any sensitivity analyses conducted to assess robustness of the synthesized results. | 6 |
| Reporting bias assessment | 14 | Describe any methods used to assess risk of bias due to missing results in a synthesis (arising from reporting biases). | 5-6 |
| Certainty assessment | 15 | Describe any methods used to assess certainty (or confidence) in the body of evidence for an outcome. | 7 |
| **RESULTS** | | |  |
| Study selection | 16a | Describe the results of the search and selection process, from the number of records identified in the search to the number of studies included in the review, ideally using a flow diagram. | 7 |
|  | 16b | Cite studies that might appear to meet the inclusion criteria, but which were excluded, and explain why they were excluded. | 7 |
| Study characteristics | 17 | Cite each included study and present its characteristics. | 7 |
| Risk of bias in studies | 18 | Present assessments of risk of bias for each included study. | 7 |
| Results of individual studies | 19 | For all outcomes, present, for each study: (a) summary statistics for each group (where appropriate) and (b) an effect estimate and its precision (e.g. confidence/credible interval), ideally using structured tables or plots. | 7-8 |
| Results of syntheses | 20a | For each synthesis, briefly summarise the characteristics and risk of bias among contributing studies. | 7-8 |
|  | 20b | Present results of all statistical syntheses conducted. If meta-analysis was done, present for each the summary estimate and its precision (e.g. confidence/credible interval) and measures of statistical heterogeneity. If comparing groups, describe the direction of the effect. | 7-8 |
|  | 20c | Present results of all investigations of possible causes of heterogeneity among study results. | 8 |
|  | 20d | Present results of all sensitivity analyses conducted to assess the robustness of the synthesized results. | 9 |
| Reporting biases | 21 | Present assessments of risk of bias due to missing results (arising from reporting biases) for each synthesis assessed. | 7, 9-10 |
| Certainty of evidence | 22 | Present assessments of certainty (or confidence) in the body of evidence for each outcome assessed. | 10 |
| **DISCUSSION** | | |  |
| Discussion | 23a | Provide a general interpretation of the results in the context of other evidence. | 11 |
|  | 23b | Discuss any limitations of the evidence included in the review. | 12-13 |
|  | 23c | Discuss any limitations of the review processes used. | 12-13 |
|  | 23d | Discuss implications of the results for practice, policy, and future research. | 13 |
| **OTHER INFORMATION** | | |  |
| Registration and protocol | 24a | Provide registration information for the review, including register name and registration number, or state that the review was not registered. | 4 |
|  | 24b | Indicate where the review protocol can be accessed, or state that a protocol was not prepared. | 4 |
|  | 24c | Describe and explain any amendments to information provided at registration or in the protocol. | 6 |
| Support | 25 | Describe sources of financial or non-financial support for the review, and the role of the funders or sponsors in the review. | 14 |
| Competing interests | 26 | Declare any competing interests of review authors. | 14 |
| Availability of data, code and other materials | 27 | Report which of the following are publicly available and where they can be found: template data collection forms; data extracted from included studies; data used for all analyses; analytic code; any other materials used in the review. | 14 |

*From:*  Page MJ, McKenzie JE, Bossuyt PM, Boutron I, Hoffmann TC, Mulrow CD, et al. The PRISMA 2020 statement: an updated guideline for reporting systematic reviews. BMJ 2021;372:n71. doi: 10.1136/bmj.n71. This work is licensed under CC BY 4.0. To view a copy of this license, visit <https://creativecommons.org/licenses/by/4.0/>

**Appendix I. PRISMA 2020 for Abstracts Checklist**

| **Section and Topic** | **Item #** | **Checklist item** | **Reported (Yes/No)** |
| --- | --- | --- | --- |
| **TITLE** | | |  |
| Title | 1 | Identify the report as a systematic review. | YES |
| **BACKGROUND** | | |  |
| Objectives | 2 | Provide an explicit statement of the main objective(s) or question(s) the review addresses. | YES |
| **METHODS** | | |  |
| Eligibility criteria | 3 | Specify the inclusion and exclusion criteria for the review. | YES |
| Information sources | 4 | Specify the information sources (e.g. databases, registers) used to identify studies and the date when each was last searched. | YES |
| Risk of bias | 5 | Specify the methods used to assess risk of bias in the included studies. | YES |
| Synthesis of results | 6 | Specify the methods used to present and synthesise results. | YES |
| **RESULTS** | | |  |
| Included studies | 7 | Give the total number of included studies and participants and summarise relevant characteristics of studies. | YES |
| Synthesis of results | 8 | Present results for main outcomes, preferably indicating the number of included studies and participants for each. If meta-analysis was done, report the summary estimate and confidence/credible interval. If comparing groups, indicate the direction of the effect (i.e. which group is favoured). | YES |
| **DISCUSSION** | | |  |
| Limitations of evidence | 9 | Provide a brief summary of the limitations of the evidence included in the review (e.g. study risk of bias, inconsistency and imprecision). | YES |
| Interpretation | 10 | Provide a general interpretation of the results and important implications. | YES |
| **OTHER** | | |  |
| Funding | 11 | Specify the primary source of funding for the review. | YES |
| Registration | 12 | Provide the register name and registration number. | YES |

*From:*  Page MJ, McKenzie JE, Bossuyt PM, Boutron I, Hoffmann TC, Mulrow CD, et al. The PRISMA 2020 statement: an updated guideline for reporting systematic reviews. BMJ 2021;372:n71. doi: 10.1136/bmj.n71. This work is licensed under CC BY 4.0. To view a copy of this license, visit <https://creativecommons.org/licenses/by/4.0/>
